# ‘You have a little human being kicking inside you and an unbearable pain of knowing there will be a void at the end’: A meta-ethnography exploring the experience of parents whose baby is diagnosed antenatally with a life limiting or life-threatening condition

**DOI:** 10.1177/02692163231172244

**Published:** 2023-05-02

**Authors:** Michael J Tatterton, Megan J Fisher

**Affiliations:** 1 School of Nursing and Healthcare Leadership, Faculty of Health Studies, University of Bradford, Bradford, UK; 2 Bluebell Wood Children’s Hospice, North Anston, Sheffield, UK; 3 International Children’s Palliative Care Network, c/o Together for Short Lives, Bristol, UK

**Keywords:** Antenatal diagnosis, family centred care, family nursing, hospice care, maternal-child nursing, palliative care, perinatal care, advance care planning

## Abstract

**Background:**

Parents of babies diagnosed with life limiting conditions in the perinatal period face numerous challenges. Considerations include the remainder of the pregnancy, delivery of the baby and decisions around care in the neonatal period.

**Aim:**

To increase understanding of how parents experience the diagnosis of a life-limiting or life-threatening condition, during pregnancy and following the birth of their baby, by answering the question: ‘what is known about the perinatal experiences of parents of babies with a life-limiting or life-threatening diagnosis?’

**Design:**

A meta-ethnography was conducted to synthesise findings from existing qualitative evidence.

**Data sources:**

British Nursing Database, CINAHL, Medline, PsycINFO and Embase databases were searched in January 2023.

**Findings:**

Relationships between parents and their families and friends, and with professionals influence the needs and experiences of parents, which oscillate between positive and negative experiences, throughout parents’ perinatal palliative care journey. Parents highlighted the need for control and a sense of normality relating to their parenting experience. Validation was central to the experience of parents at all stages of parenthood. Relationships between the parent and the baby were unwavering, underpinned with unconditional love.

**Conclusion:**

Professionals, family members and friendship groups influence the experience, validating parents and their baby’s identity and supporting parents in having a sense of control and normality by demonstrating empathy, and providing time and clear communication.

**What is already known about the topic?**

- The care and support parents and their baby receive during pregnancy, after birth and in bereavement can have a profound impact on parents and other family members.
- The diagnosis of a palliative care condition can transform a pregnancy experience, resulting in grief, anxiety, fear of abandonment, anger, hopelessness and guilt.
- The way in which professionals communicate with parents has a direct impact on the way individuals interpret information, the decisions they make and their overall palliative care experience.

**What this paper adds**

- Validation at all stages of parenthood, and of their baby’s’ personhood is central to the experience of parents.
- Parents strive to achieve a state of ‘normality’ whilst pregnant, and during and after birth. Professionals enable this though clear communication, being empathic, validating parent’s experiences, promoting dignity and being the sensitive gatekeepers of time.
- Effective, therapeutic relationships empower parents to be advocates for their baby, giving them a sense of control and identity.

**Implications for practice, theory or policy**

- Practitioners should support parents to gain a sense of control and validate their experiences as a parent during pregnancy and following the birth of their baby.
- Future research should explore the use of advance care planning from professional perspectives, to identify the barriers, enablers and impact of advance planning for multiagency professionals.
- Further research is needed on parent’s experiences of advance care planning, to identify the impact at all stages of care, and on bereavement outcomes following the death of a baby.

## Introduction

The anticipation of welcoming a new baby is usually a time of great happiness for parents and families. However, when a life-limiting or life-threatening condition is recognised or diagnosed antenatally, the reality can be very different. The support parents receive from family, friends and professionals involved in their care whilst pregnant and through the care, death and in bereavement can have a profound impact on individual family members, and on the wider family unit.^1^

Internationally, neonatal mortality rates average 17 deaths per 1000 live births.^2^ In the United Kingdom there are 2.7 deaths per 1000 live births^3^ with the leading causes of infant death including chromosomal abnormalities, congenital malformations and deformations. In 2019, over one-third of neonatal deaths were due to congenital anomalies which were incompatible with life,^4^ and therefore likely to benefit from palliative care.^5^

The diagnosis of a life-limiting or life-threatening condition can transform a pregnancy experience, resulting in grief, anxiety, fear of abandonment, anger, hopelessness and guilt.^6^ Difficult decision making is required, leaving parents feeling uncertain regarding the impact of those choices on their baby (used as an inclusive term to describe foetuses, neonates and infants) their family more widely. In order to enable informed consent and decision making, professionals should discuss the full range of treatment options, including elective termination of pregnancy, foetal intervention and palliative care.^7,8^ The way in which professionals communicate with parents has a direct impact on the way parents interpret the information and the decisions they make.^9^

Parents who decide to continue with their pregnancy face an unknown journey with uncertain ends.^10^ Considerations include the remainder of the pregnancy, birth of the baby and decisions around their care.^11^ The circumstances of individual families must be acknowledged when planning care throughout pregnancy, as well as during and following birth.^12^ Tools such as the perinatal pathway for babies with palliative care needs^5^ exist to enable professionals to provide sensitive support to parents and their babies, encouraging interdisciplinary working, and enabling timely and responsive care provision during such a distressing and uncertain time.

Perinatal palliative care is designed to enable families to spend time with their baby, with an emphasis on family centred care,^13^ and the anticipation and management of distressing symptoms.^14^ There are four broad categories of perinatal life limiting and life-threatening conditions, outlined in Table 1.

**Table 1.** Categories of perinatal life limiting and life-threatening conditions.

Category 1	An antenatal or postnatal diagnosis of a condition that is not compatible with long-term survival, for example, bilateral renal agenesis, anencephaly.
Category 2	An antenatal or postnatal diagnosis of a condition that carries a high risk of significant morbidity or death, for example, severe bilateral hydronephrosis and impaired renal function.
Category 3	Babies born at the margins of viability, where intensive care has been deemed inappropriate.
Category 4	Postnatal clinical conditions with a high risk of severe impairment of quality of life and when the baby is receiving life support or may at some point require life support, for example, severe hypoxic ischaemic encephalopathy.
Category 5	Postnatal conditions that result in the baby experiencing ‘unbearable suffering’ in the course of their illness or treatment, for example, severe necrotising enterocolitis, where palliative care is in the baby’s best interests.

Source: NHS England.^15^

The perinatal palliative care journey can be a complex one. Uncertainty around the prognosis in both the antenatal and neonatal period can occur, where the baby’s condition may change suddenly. This can lead to diversions in care throughout the perinatal journey, resulting in parents feeling confused and anxious, particularly when this is not communicated well by professionals.^9^ Maternal health is another facet to consider during this uncertain time. There is mounting evidence to suggest that there is higher incidence of serious physical and mental health needs of mothers of children who have a life-limiting condition, including an increased risk of mortality.^16–18^ Palliative care services are unequally accessed,^19^ particularly by those from minoritised groups and those who are socially disadvantaged,^20^ suggesting that there are barriers to care for parents who may have complex social or cultural needs, language barriers, neurodivergency or trauma histories. These barriers and additional complexities can further compound feelings of distrust, uncertainty and fear in parents.^21^ It is also important to consider that the perinatal palliative care journey is not only experienced by parents but also other significant family members, including a baby’s siblings and grandparents.^22^

Previous systematic reviews have considered specific elements of perinatal care, such as communication,^23^ professional partnerships^24^ and decision making^25^ following the diagnosis of a life limiting or life threatening condition. None of these reviews has focussed on the broader experience of parents during the perinatal period. We believe this is the first review to consider the experience of parents of babies diagnosed with a life-limiting or life-threatening condition in the perinatal period, to better understand the experience of parents and to identify constructs that may support the development of theory related to family-centred, perinatal palliative care.

## The review

### Aim

The aim of this review was to increase understanding of how parents experience the antenatal diagnosis of a life-limiting or life-threatening condition during pregnancy and following the birth of their baby. It was designed to answer the question: ‘*what is known about the perinatal experiences of parents of infants with a life-limiting or life-threatening diagnosis?*’

### Selecting meta-ethnography and getting started

A meta-ethnographic approach^26^ has been used, to enable interpretation, rather than simply aggregation of findings. The meta-ethnography is presented in accordance with the eMERGe reporting guidelines.^27^ This method was chosen as it enables a greater understanding of a social phenomenon through the findings of qualitative studies, yet preserving the context of individual experience, through a seven-stage synthesis process, illustrated in Table 2.

**Table 2.** Process of a meta ethnography.

Phase 1: *Getting started*	A scoping literature search identified the complexity of the perinatal experience of parents whose babies are diagnosed with a life limiting condition antenatally.
Phase 2: *Defining the area that is relevant to the initial interest*	A systematic literature search was undertaken to identify relevant studies, which were quality appraised by both authors.
Phase 3: *Reading the studies*	Studies were ready to identify the findings of each study, along with key information about each study, including sample size, methods and location.
Phase 4: *Deciding how the studies are related*	A list of themes, phrases, ideas and concepts from each paper was created. Consensus of both authors was reached on the relationship between studies, with a focus on reciprocal and refutational relationships, and potential lines of argument.
Phase 5: *Translating the studies into each other*	Having identified the key themes following the initial reading of the papers, papers were re-read and discussed to determine the presence or absence of the key themes.
Phase 6: *Synthesising translations*	Reciprocal and refutational translations were confirmed and themes were agreed.
Phase 7: *Expressing the synthesis*	The four themes of validation, dignity enablement and acceptance and hope were expressed and discussed in this article.

### Search strategy

**Figure 1.**
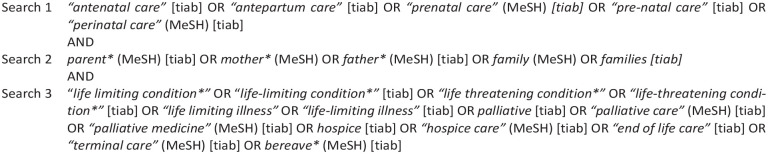
Search terms.

Systematic searching of five databases (British Nursing Database, CINAHL, Medline, PsycINFO and Embase) was conducted in January 2023. The search strategy was developed with input from a medical librarian. Search terms which were developed based on the work of Rietjens et al.,^28^ using a PICO strategy are shown in Figure 1. Due to the scant availability of published literature, broad inclusion criteria were used. Studies included in the meta-ethnography:

- Utilised qualitative methodological design, which explore the experiences of parents.
- Are primary research studies.
- Explore the experiences of parents where life-limiting or life-threatening conditions are diagnosed in their unborn babies.
- Contained verbatim quotes from participants.

The following exclusion criteria were applied to the search:

- Papers in findings written in languages other than English, to prevent translation bias.
- Studies that exclusively explored the experiences of professionals.

### Study selection and quality appraisal

A total of 1131 papers were identified from systematic searching; one further paper was identified from hand searching. Titles and abstracts were screened by both authors. Full texts of identified studies were obtained and each paper evaluated for inclusion.

**Figure 2.**
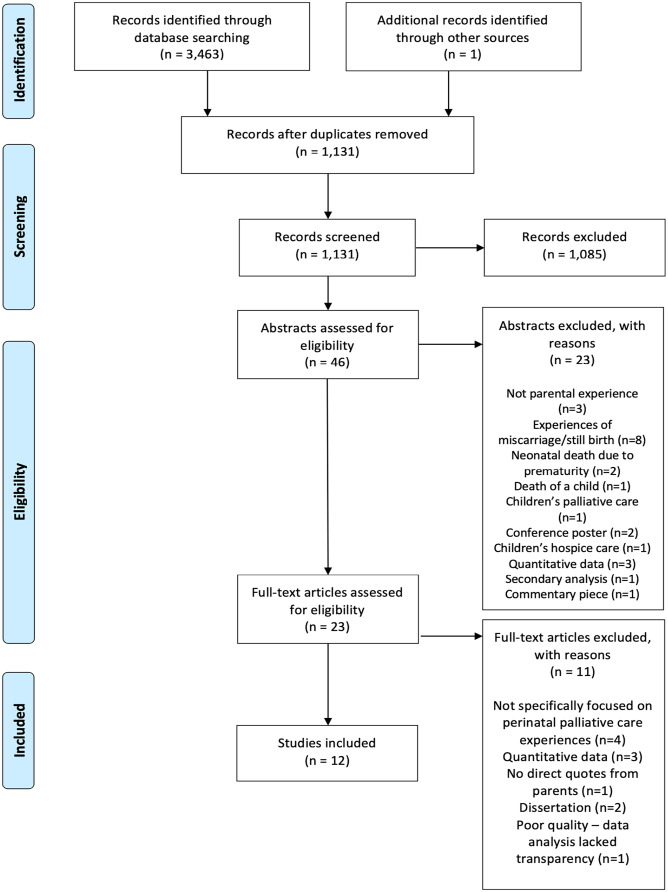
PRISMA statement.

All papers were read and assessed for relevance and quality by both authors. Papers were critically appraised using the Critical Appraisal Skills Programme qualitative research checklist^29^; scores are included in the data analysis table (Table 3). Quality appraisal led to the elimination of one paper, due to a lack of transparency around data analysis. Twelve papers from 10 studies, published between 2011 and 2022 were included in the synthesis; reasons for exclusion are detailed in the PRISMA statement, shown in Figure 2.

**Table 3.** Included studies.

Author and location	Aims	Sampling method and participants	Participant demographics	Design	Data collection and analysis	Main findings	Quality (CASP)
Côté-Arsenault and Denney-Koelsch (2011) *United States*	Explore parent’s experiences of a pregnancy with a lethal foetal diagnosis. Demonstrate feasibility and acceptability of participation of couples during such a distressing time.	Convenience sampling 5 mothers 3 fathers	Ethnicity Caucasian (7), Dual heritage (1) Age 19–38 Education All had high school education, graduate education (4) Income $0–$120,000	Qualitative descriptive design	Semi-structured, open-ended interviews Thematic analysis	- Grieving multiple losses - Arrested parting - ‘My Baby is a person’ - Fragmented healthcare - Disconnected family and friends - Utterly alone	10/10
Lathrop and VandeVusse (2011) *United States*	Explore experiences of women who chose to continue a pregnancy affected by lethal foetal diagnosis. To develop knowledge useful to healthcare professionals who provide palliative care.	Purposive sampling 15 mothers	Ethnicity African-American (1), Caucasian (13), Latinx (1) Age - 18–25 (1), 26–30 (4), 31–35 (4), 36–40 (5), 41+ (1) Relationship status - Single (1), married to father (14) Education Did not respond (1), High school or equiv (1), some college (3) technical school graduate (1), UG degree (4), graduate degree (5) Income Student (2), $46,000–$60,000 (1), $61,000–$80,000 (1), $80,000+ (7)	Narrative analysis	Open-ended interviews (1) Passages assigned to nodes (2) Comparison of nodes (3) Detailed analysis of clusters – paraphrasing and affinity groups (4) Expert and participant member checking of the above	Time: - continuity: thoughts about their babies, feelings of loss and pain, feelings of connection and ongoing feelings of grief - evolving changes: changes occur over time in thoughts and feelings, decreasing intensity of grief, personal growth and ambivalence about feelings of resolution - transient phases – connecting to past and present	10/10
Lathrop and VandeVusse (2011) *United States*	Explore the experience of perinatal hospice mothers to gather knowledge for health professionals and guide future research.	As above	As above	As above	As above	- Motherhood - Invalidation: by professionals and family/friends - Validation to the self: memento’s, photos, naming the baby - Validation through caregiving: dressing, cuddling, snuggling, kissing – connection – Validation from others: friends and family acknowledging baby - Validation from health care professionals: language, birth plans, offered choice, support	10/10
Branchett and Stretton (2012) *United Kingdom*	To determine parents’ experiences of neonatal palliative care and how this new knowledge could improve experiences of families in the future.	Purposive sampling 54 mothers 3 fathers	No demographic data collected	Qualitative survey	Qualitative responses to 2 questions posted on the SANDS forum Thematic analysis	- Importance of memory making - Empathy - Time and space - Practical help and understanding - Sensitivity - Communication with parents - Accurate documentation - Support postnatally	7/10
Côté-Arsenault et al. (2015) *United States*	To describe parents prenatal parenting after diagnosis of a life-limiting foetal diagnosis	Purposive sampling 16 mothers 13 fathers 1 partner	Ethnicity African-American (5), Asian/Pacific Islander (1), Caucasian (21), Latinx (3) Age 22–42 (mothers), 21–49 (fathers/partner) Relationship Married (11), single (3), partnered (2) Education 12–21 years (mothers), 10–19 years (fathers/partner) Income $0–$120,000 (mothers), $60,000–$80,000 (fathers/partner) Religion Christian (12), Muslim (1), other (1), none (3)	Longitudinal, phenomenological design	Open-ended, indepth interviews Cross-case analysis Member checks undertaken	-Antecedents to parenting: continuation of the pregnancy - Taking care – decisions and actions in the best interest of the baby - Promoting baby’s personhood - Interacting with baby - Being with baby - Loving baby	10/10
Côté-Arsenault et al. (2016) *United States*	To prospectively describe parents’ lived experiences of continuing a pregnancy with a lethal foetal diagnosis and to investigate the developmental tasks of pregnancy that parents undertake after the diagnosis of a life-limiting foetal diagnosis and continuation of pregnancy	As above	As above	As above	As above	- *‘Having no regrets’* - Pre-diagnosis: time of joy and positive anticipation - Learning the diagnosis: information seeking - Developmental tasks of pregnancy – relationships, comprehending implications of the condition, revised goals of pregnancy, time - Living with the diagnosis: understanding the condition and planning - Preparing for birth and death: advocating for baby with integrity - Post death: adjusting to life without a baby, grief, memorial, return to work	10/10
Fleming et al. (2016) *Switzerland*	To illuminate contemporary treatment associated with receiving a diagnosis in the antenatal period that indicates incompatibility with life. (Includes parent and professional views – professionals views are not included in this synthesis)	Convenience sampling 17 mothers 1 father 7 couples	No demographic data provided	Qualitative design	Semi-structured interviews Thematic analysis	- Shock - Choices and dilemmas - Taking responsibility - Decision to birth/death - Still pregnant: time, information, antenatal care, preparation for birth and death, support - Forming a relationship with baby - Afterwards: letting go	9/10
Cortezzo et al. (2019) *United States*	To better understand the use and experiences of birth plans after a life-limiting diagnosis (Includes parent and professional views – professionals views are not included in this synthesis)	Convenience sampling 20 parents	Ethnicity Black heritage (1) Caucasian (19) Age (mean) 31.1 (mothers), 32.9 (fathers) Education (mother) Less than high school (1), high school (2), some college/technical school (4), 2 year degree (2), 4 year degree (8), postgrad (3) Religion Christian (8) Catholic (4) NA/non/atheist (8)	Exploratory mixed methods design	Mixed-methods, exploratory, descriptive survey Thematic analysis	- Sense of control - Therapeutic - Memory making -Effective communication - Feeling prepared - Unexpected events	9/10
O’Connell et al. (2019) *Ireland*	Increase the understanding of lived experience of mothers with a prenatal lethal diagnosis who continued their pregnancy and their response to care	Convenience sampling 4 mothers	Ethnicity Caucasian (Irish) (4) Age 31–37 years old (at time of diagnosis) Religion Religious beliefs (2), atheist (1), non-practising believer (1).	Interpretative phenomenological analysis (IPA)	Semi-structured interviews IPA analysis	- Emotional impact on mother - Decision making process - The evolving relationship with baby - Experiences that hurt and experience that helps - Lasting impact	9/10
Weeks et al. (2020) *Australia*	Explore healthcare professional and parents’ views and experiences surrounding continuation of pregnancy after a ‘lethal foetal abnormality’ (Includes parent and professional views – professionals views are not included in this synthesis)	Purposive sampling 7 mothers 4 fathers	No demographic data provided	Phenomenological design	Semi-structured interviews Thematic analysis	- Variable and flexible care - Planning - Communication, uncertainty and terminology - Consistent support for perinatal palliative care key care concepts - Existence of goodwill and good intentions	9/10
Crawford et al. (2021) *United States*	Explore the experiences of women who received support from a perinatal palliative care programme after a life-limiting foetal diagnosis	Purposive sampling 12 mothers	No demographic data.	Qualitative descriptive approach	Semi-structured interviews Qualitative content analysis	- Memorabilia to cope with death and documentation of pregnancy - Acceptance of death as part of the pregnancy experience - Continued life without a child - Importance of empathy throughout the process	10/10
Hein et al. (2022) *Germany*	Explore experiences and needs of parents, reconstruct their care pathways and identify requirements for a perinatal palliative care programme.	Purposive sampling 11 mothers 9 fathers	Age 32–52 (mean 42) (mothers), 30–63 (mean 46) (fathers) Relationship Married (8), unmarried (3) Education Vocational school (mothers 3) (fathers 1), University (mothers 8) (fathers 6), Postgrad (father 2) Religion Buddhist (mother 1), Catholic (mother 5) (father 6), Protestant (mothers 3) (father 1), NA/none/atheist (mothers 2) (fathers 2)	Qualitative design	Semi-structured, narrative-oriented interviews Saldaña’s Coding Method	- Pre-natal diagnosis - The care-gap - Decision-making: Let my child decide - Parental needs and care pathways during pregnancy and birth - Parenting: spending time with the child - Bereavement	10/10

### Reading the studies

#### Data abstraction and synthesis

Synthesis began with the earliest published paper, progressing in chronological order of publication. Data were extracted from papers electronically and tabulated. No specialist software was used. Findings and discussion sections of included papers were line by line coded, developing descriptive themes. Reciprocal translation enabled the comparison of themes across papers. Refutational analysis^23^ was undertaken to explore differences, exceptions, incongruities in the accounts of experiences across papers,^30^ however no such refutation was identified.

Thematic representation was drafted independently then agreed and refined by both authors. Theoretical connections between papers are demonstrated in superordinate themes, which describe the experience of parents of baby’s diagnosed antenatally with a life-limiting or life-threatening condition. During analysis, a continuous comparison strategy was utilised to identify relationships between data. These included data that supports, contradicts or expands the interpretation of previously identified themes. Themes were discussed and agreed, ensuring traceability of the development of themes.

### Determining how the studies are related

Both authors identified key themes independently. Key findings from each study were juxtaposed to determine interstudy relationships, with a particular focus on developing themes and related quotations from study participants.

### Translating the studies into each other

Understanding of the experiences of parents was developed by translating studies into each other. Reciprocal translations were identified through the development of a table which explored cross-study comparison of the key themes, shown in Supplemental Table 1. The final synthesis created a new representation of the understanding of the experience of parents of babies diagnosed antenatally with a life limiting or life-threatening condition.

## Findings

### Expressing the synthesis

**Figure 3.**
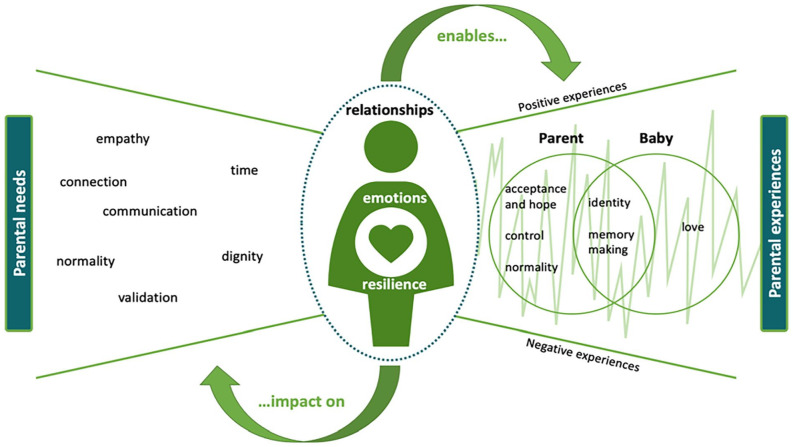
Conceptual model of the perinatal experience of parents.

Thematic analysis led to the development of a conceptual model that represents how concepts and ideas from included studies were related. Discussion of alternative explanations between authors challenged individual interpretations, consolidating the development of the conceptual model. The synthesis is presented graphically in Figure 3, although it is important to acknowledge this is not a linear journey and that parental needs and experiences fluctuate during the stages of parenthood, and with time. This expresses the main findings of our synthesis, illustrating the relationship between themes, and how they capture the experiences of parents.

## Relationships

### The parent-baby relationship

The relationship and connection between the parent and baby was at the very heart of all papers included in the synthesis.^31–42^ Even when parents felt intense, often complex emotions during their pregnancy,^31,38,40,41^ ‘. . . *I describe it as a roller coaster in the dark*’ (p. 27),^40^ all went on to love their baby unconditionally, irrespective of their diagnosis and short lives, and regardless of any other external factors, ‘*It was unbelievably painful but the most healing experience imaginable. She is a vibrant part of our family*’ (p. 15).^38^ Their babies became an integral part of families whilst they were in-utero and left a life-long impression of love on parents after they had died. This was achieved through making the most of their time together, bonding and making memories,^31–42^ ‘*Then, we just celebrated her birthday each day (. . .) There was cake and ice cream every day*’ (p. 9).^41^

Parents sought physical closeness and connectedness to the babies in the antenatal and postnatal period, demonstrated through talking to and about their baby and caring for them after death.^31–42^ Parents wanted to care for their baby, not only physically but also through advocating for them, ensuring they had the best care, and promoting their personhood.^33,36^ They wanted their baby to know that they were loved unconditionally, valued and would never be forgotten, the assurance of which provides comfort to parents both before and after death.^32–34,37^ Time and space to spend the last moments with their baby was a source of comfort at the end of life – ‘*We mostly had him in the room. We always talked to him as if he was living. To talk to our son was the* first *step in the separation*’ (p. 26).^35^

Although relationships between the parent and baby were central to the experience of parents, the relationships between the parents and their families and friends, and with professionals influenced and shaped the needs and experiences of parents. These relationships resulted in an oscillation between both positive and negative experiences, within the parents’ perinatal palliative care journey.^31–42^

### Relationships with family and friends

Relationships with family and friends were seen by some as invaluable, however navigating these relationships was acknowledged as being a source of discomfort and even loss, with others not knowing how to respond parent’s decisions to continue a pregnancy, knowing the baby has a life-limiting or life-threatening condition, or how to broach the cultural taboo of death.^31–42^ Having connections with other parents who had gone through similar experiences was affirming and validating^34,37–40^ however, this was not available to all parents: ‘*There’s hardly any forums where people exchange information. . .*’ (p. 26).^34^ Loneliness, social isolation and disconnection was experienced by parents who decided to continue with their pregnancy.^31,39^

Connectedness to others: other children, family, friends and other parents played a part in parent’s resilience and emotional wellbeing.^32,35,37^ For some, spirituality and religion assisted their progression and feelings of hope for their future without their child^35,38–41^ – ‘*Hope is now hope for something better. Hope for a life after death. Hope that heaven is closer than we think. And I feel that and I know that*’ (p. 407).^37^ Parents expressed that the lack of emotional literacy and acceptance of death by society was something that lead to difficulties in understanding the social etiquette around ‘*mourning, grieving and honouring the deceased*’ (p. 407).^37^ One mother finally felt validation for herself and her baby when she met another bereaved mother – ‘*I don’t have to act embarrassed that this thing happened and push it under the rug like it didn’t happen. It happened*’ (p. 407).^37^ One mother donated her breastmilk as a way of ‘*paying homage*’ to her son’s life.^34^

### Relationships with professionals

The therapeutic relationship between parents and professionals was fundamental; a space of support, comfort, guidance and direction for families during a challenging and uncertain time.^31–42^ Parents’ connection with professionals were strengthened through experiences of empathy, effective communication and being given time: time to process, to make informed decisions, to plan, to make memories and to grieve.^31–42^ This in turn gave parents the opportunity to deepen their relationship with their baby through bonding, memory making, love and validating their identity as a pregnant person, parents and a baby^31–42^ – ‘*So we gave her the bath, we put her hat on, we put this little gown on her, and it was. . . nice to sort of feel like. . . you know, as a mother you want to take care of your baby. But when your baby dies. . . You’re not going to feed your baby and you’re not going to get to do. . . all those things you do. . . when your baby is healthy and you bring it home. . . So to give her a bath and to dress her was really important to us*’ (p. 261).^39^ The relationship between the professional and the baby was also significant,^31–34,37,42^ offering parents comfort and assurance that their baby’s needs were met, that they were treated with dignity and respect, and were ‘*not just another statistic*’ (p. 42).^32^

Parents felt empowered when they had a sense of control and normality during the antenatal and postnatal period.^31–42^ Professionals played a significant role in enabling parent’s sense of control and feelings of normalcy through their communication, involving parents in routine antenatal and postnatal care and their expressions of empathy.^31–42^ Parents appreciated being given timely, accurate and transparent information to support their decision making and preparation for the future: ‘*It was good to know what to expect. . .we liked knowing what was going to* happen’ (p. 1342).^36^ There is recognition that the perinatal palliative care plans gave parents the opportunity to discuss the death of their baby in a safe and timely manner, ultimately leading them to a greater feeling of control.^36,37^ Although care and birth planning was expressly valued by parents as a means of taking back some control, their use was varied.^36,37^ One parent voiced how the birth plan evoked feelings of ‘*misery*’, a sense of finalisation.^34^ Although parents wanted to be kept informed of all ‘difficult’ information,^32,34,40,42^ they also valued professionals who embodied empathy. Empathic approaches to care and communication were repeatedly discussed and significantly impacted upon parents’ experiences – ‘*When she (counsellor) came, I felt like when she was there, I was safe. I felt so alone, except for when she was there. I feel like she knew what to do and what was happening and what was going on*’ (p. 408).^37^

Poor communication skills inadvertently caused distress and potential harm to parents: *‘During the scan, he [obstetrician] said: “there is nothing from here up”, as he indicated from his own brow, upwards, I couldn’t take it in*’ (p. 15).^38^ Strong communication between professionals and accurate documentation was also recognised by parents as being important, causing untoward distress if not adhered to – ‘*Please inform all relevant people of what happened. One of the monitoring hospitals wasn’t informed and we got chaser letters – very upsetting and totally unnecessary*’ (p. 43).^32^

The opportunity to engage in routine antenatal and postnatal care such as ultrasound scans, birth preparation and exercise courses gave parents a sense of identity, normality and facilitated bonding^32–35,37^ – ‘*I guess the key points were just ultrasounds, always getting to know that he was alive and had a strong heartbeat. They recorded the ultrasound for us so I have the video on a DVD of him actually alive. That was a big key point*’ (p. 406).^37^ Inconsistency in postnatal care and follow up support was evident,^32,35^ with some parents experiencing an inadequate service which left them feeling ‘*abandoned*’ (p. 43).^32^ Parents discussed gaining a sense of control in planning their baby’s funeral, and the opportunity to celebrate, remember and pay homage to them and their short life ‘*When I walked in, I was like, “I’m at my daughter’s funeral and I’m supposed to be. . . horribly sad because she just passed away” I couldn’t quit smiling. It was perfect. It was perfect. It was just kind of like, her prom, her wedding, everything combined*’ (p. 26).^40^

Despite parent’s desire for control and normalcy, it was evident that care planning and overall care experiences after a diagnosis of a life-limiting or life-threatening condition was variable.^31–42^ Parent’s experience of care during this time can profoundly impact the rest of their lives – ‘*I had question after question fired at me what felt like minutes after (my baby) was born. It was just too much for me to handle, as it just seemed like minutes since my darling baby boy had been alive with us, but everyone else wanting to simply move on*’ (p. 42).^32^

Parents expressed appreciation for their baby being cared for with respect and dignity – ‘*She took care of him for us when we couldn’t. We felt good about that*’ (p. 7).^33^ Genuine expressions of kindness and empathy from professionals following the death of a baby were important, giving parents a sense of connectedness – ‘*they were always very sensitive, treated our child with respect. . . that was lovely. . . I can’t speak more highly of the way the [hospital] looked after our Chloe, for those two months. . .’* (p. 749).^42^

Parents embraced bonding with their baby during the antenatal period, spending as much time as they could talking, reading, laughing and playing with their baby.^31,33,34,38^ During the postnatal period, professionals were seen as the gatekeepers of time: time, which ultimately gave parents the space to make memories with their baby – ‘*I think the most important thing to me was that I got to hold him and sit with him in a private room and I wasn’t rushed into anything*’ (p. 42).^32^ Spending time with their baby, even after death, meant that parents experienced a sense of normality through dressing, bathing and holding their baby, with some parents including the wider family in these special moments; reinforcing their relationship and love ‘*Our children came (. . .) and we went for a walk. I had a basket (. . .) There we put her and covered her. Then we went with our three children to a playground near the hospital. We made some pictures, how they played. And I was sitting on a rocker with the basket beside me and thought, what would people say if they knew there is a dead baby in the basket. Crazy, right? But this is family life, right?*’ (p. 10).^41^

Memory making was strongly iterated by parents as being an important part of their experience, as their memories were all they had of their baby moving into the future.^31–42^ Professional facilitation of memory making through the antenatal and postnatal period profoundly impacted upon parents – ‘*She came and took moulds of my baby’s hands and feet and did imprints, and then she took pictures. . . so I love that. That was very important to me, and she cut off a little piece of her hair, and that was helpful too just to have an actual piece of her still*’ (p. 406).^37^ Parents had individual preferences for memory making and how they wanted to spend time with their babies.^33,34^ Advice and guidance from professionals around memory making was also graciously accepted as death was often unchartered territory for parents.^32^

Acceptance around the impending death of a baby was difficult, but not unobtainable. Parents needed to be able to access information and support to be able to start to process and prepare not only for the inevitable death of their baby, but also for the pregnancy and birth, even if this meant accessing classes other pregnant women whose babies did not have a life-limiting or life-threatening condition – ‘*You read books and even attend classes during pregnancy to help prepare to have a baby, even though that’s “normal.” Its horrendous. . .*’ (p. 43).^32^ Having the opportunity to talk about the expected death of their baby in a timely way offered parents the space to regain a sense of control. This included conversations with professionals, family members or other parents who have gone through similar experiences.^31–42^ Some parents were able to accept the diagnosis early in pregnancy,^33,34,37^ however this did not mean that the death of their baby was not painful or traumatic – ‘[I] *was not anticipating being in the moment of my child’s death and wanting to do everything to save her*. . .’ (p. 1342).^36^

## Discussion

The aim of this meta-ethnography is to review parental experience of an antenatal diagnosis of a life limiting or life-threatening condition, during pregnancy and following the birth of their baby. Twelve papers published between 2011 and 2022 met eligibility criteria for inclusion. The findings from this synthesis highlight relationships are the foundation on which experience is built on. This synthesis identifies how the complex relationships parents have with professionals, each other, their family and friendship groups impact on their perception of being a parent during pregnancy, and before and after the death of their baby. Parents love their babies and strongly value the time they have together in the antenatal and postnatal period, to bond and make lasting memories. Professionals play a role in validating parents and their baby’s identity and supporting parents in having a sense of control and ‘*normality*’ through empathic, timely, clear communication.

### Parental needs

In many of the papers included in this synthesis, parents highlighted their desire for a sense of control and empowerment at all stages of parenting.^32–37^ Family centred care, which has evolved over the last 60 years to reflect the changing social construct of family, is established as standard practice within child health provision.^22,43^ Family centred care focuses on the relationship between parents and professionals, and partnership working, where family needs and preferences are considered at all stages of care.^13,44–46^ An established and effective way of achieving this in palliative care is using an advance care plan,^47–49^ offering responsive and sensitive care which meets family’s individual needs.^32,35–37^

### Advance care plans

An advance care plan can be defined as a formal plan of care, written in partnership between parents and professionals, which comprises details of a child’s condition, their wishes and ambitions and plans to manage their condition as it professionals, including symptom management and end of life care.^50^ These encompass broad and holistic perspectives, not just clinical needs, and provide professionals to share clinical information, treatment options and expectations with parents. They provide an opportunity for professionals to gain a broader understanding of the perceptions and expectations of parents, enabling misconceptions to be addressed.^49^ It is suggested that using advance care plans leads to strong and trusting partnerships between families and professionals, resulting in improved communication and therapeutic relationships.^51^ This relationship is strengthened when parents have confidence in a professional’s ability to understand the needs of their child.^40^

Parents in the included studies shared decision-making with professionals, expressing fluctuating decision-making preferences at different stages of their journey. This is consistent with research that identified that preferences for decision-making may change over time, depending on the decision that is being made,^52^ the potential consequences and their emotional readiness at the time a decision is being made.^53^ The perceived seriousness of the decision and parents’ willingness to accept a current reality^54^ also contribute to them participating in decision-making. This demonstrates the need for professionals to establish and maintain an effective therapeutic relationship with parents and families and to take a flexible and dynamic approach to decision-making as a child’s condition progresses.

### Parental empowerment

Parental empowerment as a fundamental element of family centred care.^55^ This includes the enabling or imparting power, providing parents with a sense of control. Depending at what point a life limiting condition is recognised during pregnancy, parents may have started to consider birth plans and making decisions around birth planning. These may change significantly following the diagnosis or recognition of a life limiting condition in utero. Examples of such changes include caesarean rather than vaginal delivery, breastfeeding, resuscitation and admission to the neonatal intensive care unit. Although parents may understand the necessity of such changes,^56^ the emotional consequences of these can be complex and overwhelming.

The use of advance care planning can enable parents to achieve and maintain a sense of control throughout their baby’s life.^48^ Advance care planning should be viewed as a process rather than a one-off conversation, used to facilitate individualised family centred palliative care which leads to the establishment of an effective therapeutic relationship and enables honest and sensitive communication with parents.^57^ Such an approach leads to an understanding of the biopsychosocial needs of the family and supporting parental involvement in informed decision-making.^58,59^ Despite professional apprehensions to the contrary, parents do not view advance care planning as a means only to discuss withholding treatment or resuscitation decisions.^60^ The use of advance care plans has been linked to better bereavement outcomes, and less complicated grief.^49,61^ That said, the need for professionals to make clear recommendations regarding treatment is recognised in the literature,^62^ whilst still enabling parental involvement in decision-making is preferred.^49^

In addition to calls for control, parents highlighted the need for normality relating to their parenting experience. Advance care planning can assist in this, providing professionals with opportunities to demonstrate genuine interest in parents, their baby and their experience of parenting. The inclusion of communication basics should not be overlooked, including the value and significance of using a baby’s name to demonstrate an appreciation of a baby’s life and their importance, when communicating with parents.^57^

### What this synthesis adds

This synthesis offers insight into the experiences of parents whose baby is diagnosed antenatally with a life limiting or life-threatening condition. Despite elements of parental experience being reported individually in previous publications, the combining and synthesising of findings from primary studies, and the lines of argument created in this meta-ethnography offer a new interpretation and broader perspective of the experience of parents. This meta-ethnography demonstrates how the relationships, emotions and resilience of parents are shaped by their needs, and how these affect parental experiences. Furthermore, this synthesis demonstrates how parental needs and experiences change throughout the perinatal period, and how the quality and nature of relationships with others shape this.

### Limitations

Only studies published in English were considered for inclusion, discounting research written in other languages. In addition, the review did not distinguish between the experience of parents whose babies died in utero or were still born, and those whose babies lived for a period of time after birth. These limitations should be considered when interpreting the findings of this review, and the findings considered as stimuli for further enquiry, rather than an end in itself.

### Implications for practice and research

The review demonstrates the importance of the relationship between parents and professionals at all stages following the diagnosis of a life-limiting or life-threatening condition in the perinatal period. The authors recommend the use of advance can planning. Practitioners should support parents to gain a sense of control and validate their experience as a parent during pregnancy and following the birth of their baby. Timely and empathic communication and shared decision-making can enable control and support families to accept the reality of their situation, whilst maintaining a sense of hope, in its broadest sense.

Future research should explore the experience of parents from marginalised groups, and how culture, and spiritual beliefs influence the decision-making processes. In addition, the use of advance care planning, to identify the barriers, enablers and impact of advance planning for multiagency professionals. Further research is needed on the experience of parents where advance care plans have been used, to identify its impact at all stages of care, and on bereavement outcomes following the death of a baby.

## Conclusion

This review provides insights into the experience of parents of babies diagnosed with life limiting or life-threatening conditions during the perinatal period, who are anticipating the death of their baby while in utero, or shortly after birth. The emotions and resilience of parents, as well as the quality and nature of relationships between parents and their family, friends and professionals involved in their care shape the needs and experience of parents. Parents seek to gain a sense of control, identity, normality and validation for themselves and their baby throughout the perinatal journey. Professionals can enable positive parental experiences through the establishment of a therapeutic relationship, underpinned with empathy and clear communication, alongside opportunities for parents to engage in decision-making and planning, enhances the experience of parents during such a profoundly life-changing time.

## Supplemental Material

sj-pdf-1-pmj-10.1177_02692163231172244 – Supplemental material for ‘You have a little human being kicking inside you and an unbearable pain of knowing there will be a void at the end’: A meta-ethnography exploring the experience of parents whose baby is diagnosed antenatally with a life limiting or life-threatening condition Click here for additional data file. Supplemental material, sj-pdf-1-pmj-10.1177_02692163231172244 for ‘You have a little human being kicking inside you and an unbearable pain of knowing there will be a void at the end’: A meta-ethnography exploring the experience of parents whose baby is diagnosed antenatally with a life limiting or life-threatening condition by Michael J Tatterton and Megan J Fisher in Palliative Medicine
