# Supplementary material for: ‘You have a little human being kicking inside you and an unbearable pain of knowing there will be a void at the end’: A meta-ethnography exploring the experience of parents whose baby is diagnosed antenatally with a life limiting or life-threatening condition
Source: Palliat Med. 2023 May 2;37(9):1289–302. doi: 10.1177/02692163231172244 (PMC10548777; doi:10.1177/02692163231172244)
Supplement: sj-pdf-1-pmj-10.1177_02692163231172244 – Supplemental material for ‘You have a little human being kicking inside you and an unbearable pain of knowing there will be a void at the end’: A meta-ethnography exploring the experience of parents whose baby is diagnosed antenatally with a life limiting or  [file sj-pdf-1-pmj-10.1177_02692163231172244.pdf]

Perinatal meta-ethnography

Table S1

Paper	Quotations from participants in primary studies	Interpretations of findings offered by authors
Côté-Arsenault & Denney Koelsch (2011)	*''going through all of my emotional ups and downs. I got the bad news and then I kind of took it out on him.'' (p1305)*	Relationship (parent/parent)
	''We've given him everything we could as parents.'' (p1305)	Relationship (parent/baby),
	''Treat us normally. This is our baby and we want to enjoy the pregnancy and our time with her. Don't act as if she isn't there.'' (p1305)	Relationship (parent/professional & baby/professional), time, communication, normality
	''I really do want a memory of my baby. Regardless if he's alive, or you know., he's still my baby.'' (p1305)	Relationship (parent/baby), identity
	''I want him here as long as possible; a little longer so that I can bond with him. I felt I was bonding with him when I found that I was pregnant.'' (p.1305)	Relationship (parent/baby), time, normality
	''The person who did the ultrasound didn't talk very much. And I was a little confused, [the] lady before us came out with pictures. And then we went in and she said the ultrasound machine was broken for pictures and so I thought something was wrong then. And then she didn't really say much. She just took some measurements.'' (p1305)	Relationship (parent/professional) & communication (poor)
	''I don't think some doctors know how to communicate with patients.'' (p 1305)	Relationship (parent/professional), communication (poor), empathy
	''It's almost like I'm trying to make everyone else feel better about my circumstances because they can't wrap their brain around it.'' (p1305)	Relationships (parent/friends/family), empathy (lack of)
	''At a certain point, I don't want to hear everybody's sob story because I just want to deal with my own.'' (p1305)	Relationships (parent/friends/family), empathy (lack of), communication (poor)
	'I'll just randomly get mad because of the situation. I know people mean well when they say to relax and stay/be [sic] calm. I just get mad at them 'cause I don't see how you can be relaxed and be calm in this situation.'' (p1305)	Relationships (parent/friends/family), empathy, communication (poor)

	''She was like, maybe there will be a miracle. And I'm thinking, this isn't [a] Charlie Brown special!'' (p1305)	Relationships (parent/friends/family), empathy (lack of), communication (poor)
	''We were in limbo.'' (p1305)	Relationships (parent/friends/family) - disconnection
	''We were stuck on an island, not really knowing.'' (p1305)	Relationships (parent/friends/family) - disconnection
	''We felt deserted.'' (p1305)	Relationships (parent/friends/family) - disconnection
	''So isolated.'' (p1305)	Relationships (parent/friends/family) - disconnection
	''The hard part is that we are so [geographically] isolated [from our families].'' (p1305)	Relationships (parent/friends/family) - disconnection
	''She's trying too hard to be [helpful], which is making her not helpful at all.'' (p1305)	Relationships (parent/friends/family)
	''We were the white elephant in the room. It was the first time I realized how differently we were going to be treated.'' (p1305)	Relationships (parent/friends/family) & empathy
	''I feel like she's [my mother's] been trying to make my decisions for me. I'm going to be a mom, then I have to do what's best for my child and me.'' (p1306)	Relationship (parent/family & parent/baby)
	''It's hard for doctors. Their focus is on what needs to be done, what's wrong and how do we fix it.'' (p1306)	Relationship (parent/professional), communication, empathy
	''I wouldn't be as depressed. If they saw a little bit of hope, or showed it, not even believing it.'' (p1306)	Relationship (parent/professional), communication, empathy
	[consultant was] ''very helpful with things we can do''	Relationship (parent/professional), communication, sense of control
	[palliative care had] ''different focus...being there for us on the emotional side" (p1306)	Relationships (parent/professional), empathy, communication
	''They made me feel like they would take care of her like I would take care of her as a parent, not as they would take care of her as	Relationship (parent/professional & baby/professional), empathy, communication

	a doctor [crying], that was the most important part to me.'' (p 1306)	
	''Some people say I know how you feel or I know what you're going through, but you really don't. I understand if you've lost a child, but maybe not a baby when he's born.'' (p 1306)	Relationship (parent/family/friends), empathy
	''We've given him everything we could as parents.'' (p1305)	Relationship (parent/baby), sense of control, love, identity
	''Treat us normally. This is our baby and we want to enjoy the pregnancy and our time with her. Don't act as if she isn't there.'' (p1305)	Relationship (parent/professional & baby/professional), communication, time, normality, love, identity
	''They made me feel like they would take care of her like I would take care of her as a parent, not as they would take care of her as a doctor [crying], that was the most important part to me.'' (p 1306)	Relationship (parent/professional & baby/professional), communication, normality, love
	''I want him here as long as possible; a little longer so that I can bond with him. I felt I was bonding with him when I found that I was pregnant.'' (p1305)	Love, normality, memory making
	''I need to have hope. I know what the reality is, but I still need the little bit of hope.'' (p1305)	Hope
	''Plan for the worst; hope for it being better.' (p1305)	Hope, sense of control
	''Everything that a mom gets to do, I can't do. I get to plan a funeral. It's all I can give him. He exists.'' (p1305)	Loss of identity, validation as a baby.
	''I'm going to be a mom; then I have to do what's best for my child and me.'' (p1305)	Identity, validation as a baby.
	''Treat us normally. This is our baby and we want to enjoy the pregnancy and our time with her. Don't act as if she isn't there.'' (p1305)	Validation as a baby, validation as a parent
	"I really do want a memory of my baby. Regardless if he's alive, or you know., he's still my baby.'' (p1305)	Validation as a baby, validation as a parent.

	''It's a person; [its] important to have a name. He's part of our family'' (p1305)	Validation as a baby
	'Legitimize his life.'' (p1305)	Validation as a baby
	''I want him here as long as possible; a little longer so that I can bond with him. I felt I was bonding with him when I found that I was pregnant.'' (p1305)	Validation as a baby
	''I feel like she's [my mother's] been trying to make my decisions for me. I'm going to be a mom, then I have to do what's best for my child and me.'' (p1306)	Validation as a baby, validation as a parent
	''They made me feel like they would take care of her like I would take care of her as a parent, not as they would take care of her as a doctor [crying], that was the most important part to me.'' (p 1306)	Validation as a baby
	''We were really hopeful that there was a mistake, or at least I was. We were like, maybe it'll go away by next time. I was really confident that there wasn't really going to be any problems.''	Complex emotions, loss of identity
	''I kept thinking they put me on bedrest and now I'm out of [work] and everything was going to be fine.''	Complex emotions
	''It's just, it's weird because it's almost like right now I'm just taking it so well, that I almost think that I feel like it's not happening to me. But, I think that when I see him, and when I deliver him, it's going to be totally different. Then I will have that, I-I-I will just—I know it's my baby, and I do love the baby, but I don't feel connected to the baby.'' (p1304)	Complex emotions, loss of identity
	''You can't mourn something you haven't lost yet.'' (p1304)	Complex emotions
	''This isn't my fault. As long as I hold on to that, well, because I don't want to lose my sanity.'' (p1304)	Complex emotions
	''Really, really pretty. But then, when we found out, we just stopped. So the room is like, the paneling's done around like boom-	Complex emotions

	boom, and then just...stopped. We just stopped. It just stopped. And we don't go in the room.'' (p1305)	
	''As soon as I heard the diagnosis, I started mourning.' (p1305)	Complex emotions
	''I guess I've been getting depressed lately just thinking about it.'' (p1305)	Complex emotions
	''We're going to have to spend over half of our pregnancy knowing that this baby isn't going to live.'' (p1305)	Complex emotions
	''Elena kept asking, 'Am I doing something wrong? Is this my fault? This has got to be because of something that I did.'' (p1305)	Complex emotions
	'I didn't do anything wrong; this isn't my fault.' (p1305)	Complex emotions
	''Angry that I'm 38 years old. I can support this baby. I can insure this baby. I'm a good person.'' (p1305)	Complex emotions
	''Emotional ups and downs.'' (p1305)	Complex emotions
	''It's like we're giving this baby up for adoption, except the person who is adopting him is God.'' (p1305)	Complex emotions
	''I was a wreck. I was bawling my eyes out and I couldn't sit still for the procedure.'' (p1305)	Complex emotions
Lathrop & VandeVusse (2011) Continuity and change	*"I remember talking to [another PH mother] while I was pregnant ... and, you know, it had already been a couple years for her to pass. And she goes, "just give it time. It'll get better." And I'm like, "I couldn't even begin to comprehend how in the world could all this pain actually get better." And it does." (p26)*	Relationships (parent/other parent)
	"even the bad things, you feel guilty when you start to forget ... you go back and forth, but I think you kind of want to remember the good things and maybe not so much the bad things." (p26)	To move on – time, new normality

	"The reason why I started crying was because I was so happy. I didn't feel the pain of that loss and I felt like I was forgetting her." (p26)	To move on – Time, new normality
	"even the bad things, you feel guilty when you start to forget ... you go back and forth, but I think you kind of want to remember the good things and maybe not so much the bad things." (p26)	To move on – Time, new normality
	"It was really nice just to hold her. It got harder as time went on because obviously she was dead, so her body became more and more stiff, and ... we could tell that it was getting to be time to hand her over. But, Oh! That moment was just so hard, the moment of just handing her over to the nurse and, I think that was the, the hardest moment." (p27)	Relationship (parent/baby), time, memory making
	"I'm doing better now," (p27)	To move on – Time, new normality
	"I just felt like the more information we had, the more power we had to make our time with her as meaningful as possible." (p28)	Sense of control, communication, time, relationship (parent/baby and parent/professional)
	"His passing through our lives really brought out a lot of love in our family that remains to this day." (p28)	To move on – Time, new normality, love
	"I don't know if time heals, but I almost believe the other side of grief, like when you get healed to a point, that you start thinking of your loved one, then it's a happy thought instead of a sad. ... You can think about something fun and happy and not cry, but smile. ... That's what happens over time." (p28)	To move on – Time, new normality, love
	"The 20 week sonogram anniversary, we ... had some rose bushes put in for ... a memorial rose garden ... and we thought, "Well, let's have a rose garden blessing party on that anniversary date." And so we did that and we had over a hundred people in our front yard ... and it was just really cool ... that was really healthy for me to	Relationships, love, memory making

	celebrate that ... to celebrate her life and her memory on that date instead of thinking, "this is the day of that 20 week sonogram." (p26)	
	"I just feel like I've been freed of the grief." (p26)	Time
	"Well, I think like, with any death anyone would experience in their family, over time, the pain is—the intense pain has subsided ... but you never forget them. You don't get over it, you get past; you get through it. But you don't get over it. But then, you're able to look retrospectively back and be at peace with everything ... Until you reach that point, it's pretty difficult to talk about what you've been through ... When you're in the middle of it, you just; yeah, I'd just sit there and cry the whole time."	Time
	"I remember talking to [another PH mother] while I was pregnant ... and, you know, it had already been a couple years for her to pass. And she goes, "just give it time. It'll get better." And I'm like, "I couldn't even begin to comprehend how in the world could all this pain actually get better." And it does." (p26)	Relationship (parent/other parent), time
	"I just knew we would get through. A lot of people in this kind of situation or any kind of a terminal illness realize strength that they didn't know they had ... When you're about to lose it, you don't think you can do it, it's there again for you. And so, my faith is stronger than it ever was because I felt carried. [participant's emphasis]" (p26)	Time
	"The reason why I started crying was because I was so happy. I didn't feel the pain of that loss and I felt like I was forgetting her." (p26)	Time, normality
	"even the bad things, you feel guilty when you start to forget ... you go back and forth, but I think you kind of want to remember the good things and maybe not so much the bad things." (p26)	Time, normality

	"When I walked in, I was like, "I'm at my daughter's funeral and I'm supposed to be ... horribly sad because she just passed away." I couldn't quit smiling. It was perfect. It was perfect. It was just kind of like, her prom, her wedding, everything combined." (p26)	Love, sense of control, acceptance
	"And then you have this beautiful child and beautiful moments ... and their life, even though it was incredibly short, was so meaningful and so profound ... and knowing how proud you were of that baby ... that just really gave us a high ... where you're just happy and proud and ... very, very relieved ... And then that high, you know, it gradually goes down ... It wasn't like I was always really, really down, but there were, I describe it as a roller coaster in the dark. You ... have blinders on. And you don't know how long you'll have a medium level, when you'll have a dip, when you'll have another high" (p27)	Love, time, acceptance
	"There was so much involved in having this special baby, and every time I think back to the times when I was pregnant with her, I would do it all exactly the same. I would make all of the same decisions again. So you know, I have no regrets on everything." (p27)	Relationship (parent/baby), love, acceptance
	"We feel that [baby]'s life was not a mistake in any way, and ... we do believe that she was put here for a reason ... sometimes it's the most weak and innocent lives among us that can teach us the most." (p28)	Love, acceptance
	"I just remember being so proud of him. That is probably the biggest thing that still stands out in my mind ... I was just so proud of him. I still am," (p28)	Love
	"When someone passes through your life, they leave an imprint on you, and maybe that's what it is: maybe it's his spirit. Maybe it's an energy. Like my oldest daughter said, she has a big brother in	Love, acceptance, spirituality

	Heaven, someone looking out for her, someone watching over her." (p25)	
	"The 20 week sonogram anniversary, we ... had some rose bushes put in for ... a memorial rose garden ... and we thought, "Well, let's have a rose garden blessing party on that anniversary date." And so we did that and we had over a hundred people in our front yard ... and it was just really cool ... that was really healthy for me to celebrate that ... to celebrate her life and her memory on that date instead of thinking, "this is the day of that 20 week sonogram." (p26)	Love, acceptance, spirituality
	"I'll never be the same ... it really made me a lot more compassionate person," (p26)	Love, acceptance, spirituality
	I just knew we would get through. A lot of people in this kind of situation or any kind of a terminal illness realize strength that they didn't know they had ... When you're about to lose it, you don't think you can do it, it's there again for you. And so, my faith is stronger than it ever was because I felt carried. [participant's emphasis] (p26)	Love, acceptance, spirituality
	"We feel that [baby]'s life was not a mistake in any way, and ... we do believe that she was put here for a reason ... sometimes it's the most weak and innocent lives among us that can teach us the most." (p28)	Love, acceptance, spirituality
	"This birth is sacred. This child is a gift from God, and we want to honor his life here." (p28)	Love, acceptance, spirituality
	"And then you have this beautiful child and beautiful moments ... and their life, even though it was incredibly short, was so meaningful and so profound ... and knowing how proud you were of that baby ... that just really gave us a high ... where you're just happy and proud and ... very, very relieved ... And then that high, you know, it gradually goes down ... It wasn't like I was always	Validation as a baby

	really, really down, but there were, I describe it as a roller coaster in the dark. You ... have blinders on. And you don't know how long you'll have a medium level, when you'll have a dip, when you'll have another high" (p27)	
	"I just remember being so proud of him. That is probably the biggest thing that still stands out in my mind ... I was just so proud of him. I still am," (p28)	Validation as a baby
	"The 20 week sonogram anniversary, we ... had some rose bushes put in for ... a memorial rose garden ... and we thought, "Well, let's have a rose garden blessing party on that anniversary date." And so we did that and we had over a hundred people in our front yard ... and it was just really cool ... that was really healthy for me to celebrate that ... to celebrate her life and her memory on that date instead of thinking, "this is the day of that 20 week sonogram." (p26)	Validation as a baby, validation as someone who was pregnant, validation as a parent.
	"There isn't a day that goes by when you don't think of her," (p 25)	Complex emotions
	"it's been nine years, but we still think of him every day and every time we say a prayer, we always say 'Give [baby] a hug and a kiss.' Every day." (p25)	Complex emotions
	"I do still have just those moments when ... something snaps and I just remember that I don't have a baby, you know, and I never will have him again." (p25)	Complex emotions
	"it hasn't gotten any easier." (p25)	Complex emotions
	"I don't cry every day. Most of the time ... it's always there, but it's not overwhelming grief in our life daily," (p26)	Complex emotions
	"I just feel like I've been freed of the grief." (p26)	Complex emotions

	"Well, I think like, with any death anyone would experience in their family, over time, the pain is—the intense pain has subsided ... but you never forget them. You don't get over it, you get past; you get through it. But you don't get over it. But then, you're able to look retrospectively back and be at peace with everything ... Until you reach that point, it's pretty difficult to talk about what you've been through ... When you're in the middle of it, you just; yeah, I'd just sit there and cry the whole time." (p26)	Complex emotions
	"I remember talking to [another PH mother] while I was pregnant ... and, you know, it had already been a couple years for her to pass. And she goes, "just give it time. It'll get better." And I'm like, "I couldn't even begin to comprehend how in the world could all this pain actually get better." And it does." (p26)	Complex emotions
	"immediate crisis mode" (p26)	Complex emotions
	"We were just ... in complete shock, just complete shock, and I don't even remember how the rest of that conversation went. And I remember walking out just thinking, "Oh, my goodness, we're in a ... really bad nightmare and I'm sure we will wake up." (p27)	Complex emotions
Lathrop & VandeVusse (2011) Affirming Motherhood	*''it was a really big deal to me,'' (p259)*	Relationship (parent/professional), communication, empathy (lack of)
	''that was offensive,'' (p259)	Relationship (parent/professional), communication (lack of), empathy (lack of)
	''at that point, I got angry at, like, the medical profession in general.'' (p259)	Relationship (parent/professional), communication, empathy (lack of)
	"Well, the doctor said we have to terminate: ''It's non-compatible with life.'' … who are they to say that? You know what I mean? Who are they to take all this wonderful, beautiful experience that we had away just because they feel it's non-compatible with life?" (p260)	Relationship (parent/professional), communication (lack of), empathy (lack of)

	''we did have a different journey than it sounded like they were telling us.'' (p260)	Relationship (parent/professional), communication (lack of)
	"Anything tangible that you can get your hands on is so important. The one thing you want is already gone, and … you've got to have that piece of something for comfort." (p260)	To move on – love, memory making, new normality
	"I'm so glad I have those pictures because otherwise I'd think that really didn't happen to me …. That was just a bad dream, you know. But the pictures are … proof that the baby did exist." (p260)	To move on – love, memory making, new normality
	"I slept with him. Just held him real close to me. Talked to him a lot, kissed him a lot, just tried just to savor every moment I could … I would put him down in his little isolette … [then soon] I would pick him up again and just snuggle him real tight to me and talk to him and tell him about who loves him, and tell him about [his brother], and the things I would have wanted for him in his life. And it was like trying to live a lifetime with him." (p261)	Relationship (parent/baby), normality, love, memory making, time
	"So we gave her the bath, we put her hat on, we put this little gown on her, and it was … nice to sort of feel like … you know, as a mother you want to take care of your baby. But when your baby dies …. You're not going to feed your baby and you're not going to get to do … all those things you do … when your baby is healthy and you bring it home … So to give her a bath and to dress her was really important to us." (p261)	Relationship (parent/baby), normality, love, memory making, time
	"I got to hold her alive for nine months. I knew she was very real, very alive. I could feel her kicking … and I knew that she was a little human … a little person. But I didn't know how much my husband really felt that, and so I knew if he could hold her alive, he could have the same experience that I had gotten to have for nine months." (p261)	Relationship (parent/baby, parent/parent), normality, love, memory making

	"I said 'Do you remember me?' And [the nurse] said 'Of course, you're [baby]'s mom.' And … I'm going to go back there just because I'm only [baby]'s mom. It's my identity and I like it."	Relationship (parent/professional), communication, empathy, normality, identity
	''We needed those people to acknowledge what was going on,'' (p261)	Relationship (parents/family/friends), love
	''I think it just reconfirmed that she [the baby] was important to other people.'' (p261)	Relationship (parents/family/friends), love
	"You're still a mom, and you have a need to talk about your baby. Well, everyone else who hasn't gone through that … really feels uncomfortable around you when you start talking about your baby; wants to shut you down and change the subject … so you don't get the opportunity to talk about your baby … I've got all these pictures. To somebody in the outside world, that's gruesome … But you can come here [perinatal hospice agency] and the … moms bring their photo albums, and we can show each other our babies. And we can fulfill the need that we have to talk about our kids. That they're real people" (p262)	Relationships (parent/family/friends, parent/professional, parent/other parent), communication, empathy and memory making
	"The second perinatologist, he was so cool. He was like, ''You're right. This life is of value, and every life has a value.'' (p263)	Relationship (parent/professional), communication, empathy
	''she just validated all my feelings were okay feelings.'' (p263)	Relationship (parent/professional), communication, empathy
	''we need you to be real, and, you know, take off that white lab coat and become a human.'' (p263)	Relationship (parent/professional), communication, empathy
	"I looked at it and I was like, ''You're kidding me. This is what we're going to bury her in?'' And I got really pissed. I was just like, ''That's not even good enough … I've seen Igloo ice coolers that are nicer than that.'' (p259)	Lack of dignity
	''gift from God.'' (p259)	Acceptance, hope, love, spirituality

	"I looked at it and I was like, ''You're kidding me. This is what we're going to bury her in?'' And I got really pissed. I was just like, ''That's not even good enough … I've seen Igloo ice coolers that are nicer than that.'' (p259)	Invalidation
	"Well, the doctor said we have to terminate: ''It's non-compatible with life.'' … who are they to say that? You know what I mean? Who are they to take all this wonderful, beautiful experience that we had away just because they feel it's non-compatible with life?" (p260)	Invalidation
	"I'm so glad I have those pictures because otherwise I'd think that really didn't happen to me …. That was just a bad dream, you know. But the pictures are … proof that the baby did exist." (p260)	Validation as a baby, validation as a parent, memory making
	"Anything tangible that you can get your hands on is so important. The one thing you want is already gone, and … you've got to have that piece of something for comfort." (p260)	Validation as a baby, validation as a parent, memory making
	"You're just kind of trying to hold onto something, because everything's slipped away and that's all you have now. And so, if you're ever in doubt of what you went through, you can just go back and revisit." (p260)	Validation as a baby, validation as a parent, memory making
	''makes it real, and not a distant memory. Something that helps you recall it ... you don't want to forget them. They're still your baby'' (p260)	Validation as a baby, validation as a parent, memory making
	"I slept with him. Just held him real close to me. Talked to him a lot, kissed him a lot, just tried just to savor every moment I could … I would put him down in his little isolette … [then soon] I would pick him up again and just snuggle him real tight to me and talk to him and tell him about who loves him, and tell him about [his brother], and the things I would have wanted for him in his life. And it was like trying to live a lifetime with him." (p261)	Validation as a baby, validation as a parent

	"So we gave her the bath, we put her hat on, we put this little gown on her, and it was … nice to sort of feel like … you know, as a mother you want to take care of your baby. But when your baby dies …. You're not going to feed your baby and you're not going to get to do … all those things you do … when your baby is healthy and you bring it home … So to give her a bath and to dress her was really important to us." (p261)	Validation as a baby, validation as a parent
	"I got to hold her alive for nine months. I knew she was very real, very alive. I could feel her kicking … and I knew that she was a little human … a little person. But I didn't know how much my husband really felt that, and so I knew if he could hold her alive, he could have the same experience that I had gotten to have for nine months." (p261)	Validation as a baby, validation as a parent
	"I said 'Do you remember me?' And [the nurse] said 'Of course, you're [baby]'s mom.' And … I'm going to go back there just because I'm only [baby]'s mom. It's my identity and I like it." (P261)	Validation as a baby, validation as a parent, identity.
	''We needed those people to acknowledge what was going on,'' (p261)	Validation as a baby, validation as a parent
	"'I think it just reconfirmed that she [the baby] was important to other people.'' (p261)	Validation as a baby
	"You're still a mom, and you have a need to talk about your baby. Well, everyone else who hasn't gone through that … really feels uncomfortable around you when you start talking about your baby; wants to shut you down and change the subject … so you don't get the opportunity to talk about your baby … I've got all these pictures. To somebody in the outside world, that's gruesome … But you can come here [perinatal hospice agency] and the … moms bring their photo albums, and we can show each other our babies. And we can fulfill the need that we have to talk about our kids. That they're real people" (p262)	Validation as a baby, validation as a parent

	"The second perinatologist, he was so cool. He was like, ''You're right. This life is of value, and every life has a value.'' (p263)	Validation as a baby
Branchett & Stretton (2012)	*"One of my very favourite [photos] is one taken by one of the lovely nurses. [my baby] was in the middle of his care so he had no breathing aid, no hat, no lines, just him in a little nappy curled up, the first time I saw all his hair properly, and it means soooooooooo much to me. Seemingly small things like writing his name on it, with 'To mummy and daddy' and the time, place and date just make such a difference." (p42)*	Relationship (parent/professional, baby/parent & baby/professional), 'normality, identity, love, memory making
	"…the first day I saw him wearing clothes whilst he was still alive, was just worth more than gold and I will NEVER forget how he looked that day. Most importantly, he looked like a baby." (p42)	Relationship (baby/parent), 'normality', memory making
	"We had a chance to be in a private room and spend time with her. The nurse helped us bathe her and dress her. I'm glad they did. We will remember that time fondly. It is the only chance we got to really feel like her parents." (p42)	Relationship (parent/professional, baby/parent & baby/professional), 'normality', love, time, identity, memory making
	'(My baby) had been in NICU for nearly 3 weeks and it helped that the nurses that had cared for him in that time came and said goodbye to him. It showed me that he was not just another statistic, he was my baby' (p 42)	Relationship (parent/professional, baby/parent & baby/professional), empathy
	"...caring and compassionate staff who not only are there for the mum but for the whole family, as I think they get forgotten. We had some lovely midwives who were even crying with my mother in law. Some may say this is unprofessional, however we just felt how much they cared." (p42)	Relationship (parent/professional, baby/parent & baby/professional), empathy
	'…the tears on other faces do count…in a big way' (p42)	Relationship (parent/professional & baby/professional), empathy
	'I think the most important thing to me was that I got to hold him and sit with him in a private room and I wasn't rushed into anything.' (p42)	Relationship (baby/parent), time, memory making & 'normality'
	"I had question after question fired at me what felt like minutes after [my baby] was born. It was just too much for me to handle, as it just seemed like minutes since my darling baby boy had been alive with us, but everyone else wanted to simply move on." (p42)	Communication (poor), empathy (lack of) & time

	"My hubby being able to stay over too meant so much. (Daddies hurt too and we were going through it together.) Most of the time we were treated wonderfully and with total respect, apart from one silly woman. When I went to get my husband a cup of tea after he drove over 60 miles to see me and [my baby] after work one evening, she snapped: "Drinks are for patients only!" in a very rude way. I know it's trivial but I honestly wanted to punch her." (p42)	Relationship (parent/professional), communication (poor) & empathy (lack of)
	"Luckily my other half had to go home briefly to check on my step son and staff had the foresight to get him to pick up our digital camera." (p42)	Communication
	"...please be sensitive to parents needs after the loss. They are likely not to be thinking straight and for us, decision making was near impossible..." (p42)	Empathy & communication
	"After our daughter passed, the midwives in postnatal moved me to a different room further away from screaming babies. This was helpful and I'd recommend." (p 43)	Relationship (parent/professional) & empathy
	"Please keep parents informed. It seems a constant uphill struggle to obtain information. particularly in the hours immediately after delivery or transfer." (p43)	Communication & sense of control
	"Be honest with parents and don't be scared of telling the truth. People cope they don't have a choice." (p43)	Communication & sense of control
	"It's scary enough watching your baby fight for life, but if you are unaware of how serious things can be, then the total shock just adds to the pain..." (p43)	Communication & sense of control
	"You read books and even attend classes during pregnancy to help prepare to have a baby, even though that's 'normal'. It's horrendous, but even more important to have some help to prepare yourself for losing your precious baby. Mostly because that doesn't feel 'normal' at all and you haven't a clue what to do." (p43)	'Normality'
	"Please record what happens in the delivery room and afterwards accurately. Having to correct notes or even worse, discover that they have been lost, causes untold misery and hurt..." (p43)	Communication (poor)

	"Please inform all relevant people of what happened. One of the monitoring hospitals wasn't informed and we got chaser letters very upsetting and totally unnecessary." (p43)	Communication (poor)
	"Having a specialist midwife with us at all appointments with our consultant, really helped...she would spend time with us to explain things in Janet and John terms. This helped an awful lot and made our experiences much easier." (p43)	Relationship (parent/professional), communication & 'sense of control'
	"I didn't get any follow up support...I felt like I had been abandoned. I since know that if I had called the hospital there was a bereavement midwife but I didn't know that and I feel that she should have called me." (p43)	Communication (lack of)
	"Great community support really helped, I had several midwives come out to my home and chat for hours. I think without this support I would not have coped."(p43)	Relationship (parent/professional) & communication
	"We had a chance to be in a private room and spend time with her. The nurse helped us bathe her and dress her. I'm glad they did. We will remember that time fondly. It is the only chance we got to really feel like her parents." (p42)	Relationship (parent/professional, baby/parent & baby/professional), memory making, time, love, identity
	"Having our daughter recognised as an individual, not just part of a conveyor belt to be got out of the way, really helped. She was treated as a baby, with great respect and that meant so, so, much to us." (p42)	Relationship (parent/professional, baby/professional), dignity, empathy, identity
	'(My baby) had been in NICU for nearly 3 weeks and it helped that the nurses that had cared for him in that time came and said goodbye to him. It showed me that he was not just another statistic, he was my baby' (p 42)	Relationship (parent/professional, baby/parent & baby/professional), empathy
	"I was like, just cover [her] up like, you know, like she's fine...Yes. That meant a lot to me. I mean I didn't think about it before but I was like protective of her." (p7)	Relationship (baby/parent & parent/professional), love, identity
	"…Seemingly small things like writing his name on it, with 'To mummy and daddy' and the time, place and date…just make such a difference." (p42)	Validation as a parent, validation as a baby
	'We had a chance to be in a private room and spend time with her. The nurse helped us bathe her and dress her. I'm glad they did. We will	Validation as a parent, validation as a baby

	remember that time fondly. It's the only chance we really got to be her parents' (p 42)	
	'Having our daughter recognized as an individual, not just part of a conveyor belt to be got out of the way really helped. She was treated as a baby, with great respect and that meant so, so, much to us' (p42)	Validation as a baby
	'(My baby) had been in NICU for nearly 3 weeks and it helped that the nurses that had cared for him in that time came ands said goodbye to him. It showed me that he was not just another statistic, he was my baby' (p 42)	Validation as a parent, validation as a baby
Côté -Arsenault et al. (2015)	*"I went on the internet to kind of get a little bit more research on it. I did see some of the photos of the babies online that are diagnosed with trisomy 13.' (p6)*	Sense of control
	"Knowing that we're pulling out the stops, and leaving no stone unturned and leaving it on the field, so to speak, gives me solace" (p7)	Relationship (baby/parent), sense of control, love
	"The doctor even asked us if we had a name picked out for the baby before he told us the results. That tiny gesture right there skyrocketed my respect for him' (p8)	Relationship (parent/professional), 'normality', communication, empathy
	"Watching her move and kick is amazing, given the circumstances. Seeing her suck her thumb made everything feel so normal. I can't wait to hold her. But I've heard the heart beating – many times now. I've seen her squirming. I've felt her kicking. I know she's very much alive' (p10)	Relationship (baby/parent), identity, time, 'normality', memory making
	"I wanted her [the baby] to still be close to me like she'd be laying there over on the table. I actually slept with her in my arms the whole night" (p10)	Relationship (baby/parent), time, love, memory making and 'normality'
	"I was like, just cover [her] up like, you know, like she's fine...Yes. That meant a lot to me. I mean I didn't think about it before but I was like protective of her." (p7)	Relationship (baby/parent & parent/professional), love, identity
	"Protective about who would take his baby to the morgue. We actually asked someone that we felt close to [a nurse], to please take him and not leave him alone until they came because they were on their way and we weren't able to do it." (p7)	Relationship (parent/professional, baby/professional), empathy
	"She took care of him for us when we couldn't. We felt good about that" (p7)	Relationship (parent/professional, baby/professional)

	"I kept asking her, 'Do you have extra blankets to make him comfortable?'... because I didn't want him to be cold in the morgue" (p8)	Relationship (baby/parent), love
	"...his hand and footprints, and photos" (p8)	Memory making
	"The important part is to keep Aaron with us as much as possible because we don't want him to pass away from us basically so as much as they can do in regards to him being with us most of the time" (p10)	Relationship (parent/professional & parent/baby), memory making & time
	"Then our pastor baptized him right away in that first minute. That right there was like our primary goal with Tyler once we found out, you know, what he was diagnosed with [trisomy 18], was just to have that live birth and have him baptized. Being able to see that was really nice." (p7)	Spirituality
	"[My milk] came in and I kind of – I was kind of surprised, but then I made the decision that all this is happening for a reason. I was accepted as a donor at the...milk bank. So I have been donating there." (p8)	Acceptance
	"Our goal was to let Blake know that we loved him more than life itself and we were so honored and proud of him. I felt so bad for crying when I found out about his disease that I wanted him to know how much I do/did love him. It was never me crying about him I was crying about what was happening to him" (p8)	Love & relationship (parent/baby)
	"Her urn is in our bedroom and kind of just on display for the two of us" (p8)	Relationship (parent/baby), love
	"Well, me and Darnell talk to her all the time. We have family time every day, ask her if there's something that she wants us to do, we'll do it, you know, kick once for yes or kick twice for no. We read to her. We just laugh. We laugh!" (p9)	Relationship (baby/parent), identity, time, memory making & 'normality'
	"I'm pretty sure that Else knew his voice. When we'd crash on the couch after the other kids were in bed, she would start moving around a little bit more when he started talking. When he and I 'argued' over what to watch on TV, she kicked more when he talked about football" (p9)	Relationship (parent/baby), identity, time, memory making & 'normality'
	"Then I'd put my head down on her chest and let do my head like that with her little hands [she demonstrated the position]. I'd be like, 'Calm down, Brianna. Stop it," and she would just [whimper] and fall on off to sleep" (p9)	Relationship (baby/parent), time, identity, 'normality'

	"I'm paying close attention to her kicks and punches, so I know she is here" (p10)	Relationship (baby/parent), love, memory making, time and 'normality'
	"Watching her move and kick is amazing, given the circumstances. Seeing her suck her thumb made everything feel so normal. I can't wait to hold her. But I've heard the heart beating – many times now. I've seen her squirming. I've felt her kicking. I know she's very much alive' (p10)	Relationship (baby/parent), love, memory making & time
	"We talk to her [the baby] most all of the time. We have family time every day, she hears what's going on, and she is with us" (p10)	Relationship (baby/parent), love, memory making, time & 'normality'
	"The important part is to keep Aaron with us as much as possible because we don't want him to pass away from us basically so as much as they can do in regards to him being with us most of the time" (p10)	Relationship (baby/parent), love, memory making & time
	"We all need to do what we can and just be her parents and let her know that we are here with her" (p10)	Relationship (baby/parent), love, memory making & time
	"I just would rather her have whatever time she has here and make it the best of all of us, you know spend as much time with her as possible…Now, I do find myself, you know, putting my hand on my stomach and trying to find the movements to make sure she's still in there. I need to feel her there and I need to feel her move. I really want her to be born alive so I can hold her for just a few minutes" (p10)	Relationship (baby/parent), love, memory making & time
	"I wanted her [the baby] to still be close to me like she'd be laying there over on the table. I actually slept with her in my arms the whole night" (p10)	Relationship (baby/parent), love, memory making & time
	"It's like when it's just me and her, like we're still trying to be happy and enjoy life and we're not dwelling on the fact that he'd going to pass away and they are." (p11)	Relationship (baby/parent), love, memory making, time, 'normality'
	"He was my miracle, my joy, my greatest accomplishment. He was why I was here on the earth. I was supposed to bring him to life and introduce him to the world. I loved him the minute he was born and every day since" (p11)	Relationship (baby/parent), love & acceptance
	"My baby was perfect. She [could] move her little arms, kick her little legs. There just wasn't much of it. She could get mad. It was just so funny. That's the part that I love the most when she would just get so	Relationship (baby/parent), love, memory making, time, normality

	upset…I just want you to do everything you can. I need her. I want her. I don't want to be selfish, but I want my baby. I want her." (p11)	
	"It wasn't anything I was prepared for because everybody kept telling me that, you know, it's your baby and when you see him, he's just perfect and all this stuff and that really was it. He was just awesome!" (p11)	Relationship (baby/parent), love, identity, acceptance.
	"When we had to say goodbye to her in the hospital, John was the one who took her from my arms and placed her so gently in a basket. A nurse didn't do it. The funeral home man didn't do it. Elsa's daddy did. It took an immeasurable amount of love - for me and his Elsa - to do that…At the end of the service, I watched John walk over to Elsa's casket and pick it up. It was probably the moment he felt his weakest as a daddy. But he showed more strength and love and sacrifice in that moment than I have ever seen. As awful as it was, there was beauty in that moment." (p11-12)	Relationship (baby/parent) and love
	"The doctor even asked us if we had a name picked out for the baby before he told us the results. That tiny gesture right there skyrocketed my respect for him' (p8)	Validation as a parent, validation as a baby
	"Her urn is in our bedroom and kind of just on display for the two of us" (p8)	Validation as a parent, validation as a baby
	"I'm paying close attention to her kicks and punches, so I know she is here" (p10)	Validation as a baby
	"We all need to do what we can and just be her parents and let her know that we are here with her" (p10)	Validation as a baby
	"Oh, this is Tyler's first time at Yogi Bear [camp grounds]" (p10)	Validation as a baby
	"He was my miracle, my joy, my greatest accomplishment. He was why I was here on the earth. I was supposed to bring him to life and introduce him to the world. I loved him the minute he was born and every day since" (p11)	Validation as a baby and as a parent
Côté -Arsenault et al. (2016)	*"In the big picture, doing this … we can look back and we can say we did everything humanly possible for him, giving him every possible chance that we could for him to survive. Six months from now, if we lose him, that's a difference maker." (p103)*	Relationship (baby/parent), sense of control

	"I wanted to know what was next. You're still pregnant and still have this beautiful baby that you're expecting and now you have to know where to go from here." (p104)	Sense of control, communication
	"I still am [disappointed]. I actually kind of told him … He brought up, you know, reasons why it might be better for the baby: the NICU is there if we decide to go that route and of course, they have more experience." (p106)	Relationship (parent/professional) & communication (poor)
	"Seeing her on the ultrasound last week … and how happy that made him, but we was in there just laughing, having so much fun." (p106)	'Normality', relationship (parent/baby, baby/sibling), memory making & time
	"I don't know that it really changes much, but I think it's really wonderful. This may be the only opportunity that we actually have to meet her in all her life." (p106)	'Normality', relationship (parent/baby), time, memory making
	"It is nice especially for like me because I don't get to feel her every day. I don't have that bond and so to see her is huge for me because I need that." (p106)	'Normality, relationship (parent/baby) & time
	"I think he kind of prepped everybody." (p106)	Relationship (parent/professional), sense of control & communication
	"I realized halfway through Friday that part of my misery was just thinking about having to do this birth plan on Saturday."... "It raised a lot of questions, not that we haven't thought about it and hadn't talked about it, but I think it was finalizing it that was making it the most difficult." (p106)	Sense of control & communication
	"Chris and I went to the funeral home already and we talked it over with them like what we need to do or what happens because we have never been a part of any planning any of these things before to know what would have to happen … It was weird … I didn't think that would be on my agenda until I was about 50 or so." (p106)	Sense of control
	"Somebody is looking out for my child. And doing everything he can to help … it didn't change anything about her prognosis. But it helped, it helped me." (p106)	Sense of control, relationship (parent/professional), empathy & communication

	"She said that it's really just the whole, being the last person to touch them thing. That really resonated with me because that's really how it felt." (p107)	Sense of control, relationship (parent/professional & parent/baby), empathy & communication
	"Somebody is looking out for my child. And doing everything he can to help … it didn't change anything about her prognosis. But it helped, it helped me." (p106)	Relationship (baby/professional and parent/professional), empathy
	"She said that it's really just the whole, being the last person to touch them thing. That really resonated with me because that's really how it felt." (p107)	Relationship (baby/professional, parent/baby and parent/professional)
	"One of the hardest things throughout this journey has been breaking the news to people." (p104)	Relationships (parent/family/friends), communication
	"Part of the whole reason for us to send out the notice that we did to everybody is because we didn't want to keep rehashing the same story of what's going on and being brought down or crying about this every day" (p104)	Relationship (parent/family/friends), communication
	"I only have two people, and I don't consider them friends anymore, that when I first told them … they had a negative reaction … You go your way and I go mine and we'll just leave it like that." (p105)	Relationship (parent/family/friends)
	"We kind of have a motto, "Five minutes at a time," but we do have a lot of comfort knowing that we're doing the right thing and we're going to get to see her and meet her. We just pray that she's with us for a short time and how she can feel our touch and hear our voice." (p105-106)	Relationship (parent/baby), love, acceptance, hope
	"A couple of weeks ago, we decided we'd start reading a book each night before we go to bed, kind of liked we have our favorite children's books that our parents had kept … For me personally, I think that makes me feel more connected to Leah because they do say that they can hear you and things like that." (p106)	Relationship (baby/parent), 'normality', time & memory making
	"I promise you, I was gloriously happy. I felt his angel glow or something." (p106)	Acceptance, hope, love, spirituality
	"Tomorrow, I will see my little girl one last time, attend a service in her honor, and bury her. In some ways, I just want the day to be over. But in so many other ways, I don't want tomorrow to come at all." (p107)	Relationship (parent/baby), acceptance

	"I mean I don't think we'll ever look back and say it was too hard, we wish we hadn't done it because even though we lost Leah, this has been the most miraculous time of our lives. I mean it was just what we've learned, what we've gained, the love that we had, I mean we can't exchange that for anything." (p107)	Relationship (parent/baby), acceptance, hope & love
	"Now, I feel like I flip flopped. I went from being very private to like singing it to the rooftops like, 'Who wants to see my photos? Let me just tell you the whole day.' I've really sparsed down how much I share sometimes because it does, it opens up a lot of emotions" (p105)	Validation as a parent and validation as a baby
Fleming et al. (2016)	*"The midwives even listened to the heartbeat. They treated me like a normal pregnant woman. And that gave me a good feeling instead of being pushed away because the baby would die." (p26)*	Relationship (parent/professional) 'normality' & identity
	"[I wanted] to talk to someone who could tell me exactly how the birth would go. We also said we were not in a hurry…..and the midwife encouraged us not to be frightened." (p26)	Relationship (parent/professional), communication, empathy & time
	"She prepared us for the birth so well, just we two and her. So really I had a normal birth preparation, what is labour, how does it go and [we] only later [talked] about our specific situation and what we could do" (p26)	Relationship (parent/professional), time, 'normality' & sense of control
	"I found the information and care was very, very good. They knew what was happening and I don't think we missed out on anything. We always knew what options we had and we asked a lot too" (p26)	Relationship (parent/professional), communication
	"You know, it was really difficult for me. To know how someone could help me. You've been to hell and back and now after five weeks in the hospital there is no one to help" (p26)	Relationship (parent/professional), communication (poor)
	"I found it really good to be in a course with other parents who'd had more or less the same experience. I can recommend that, it really helped" (p27)	Relationship (parent/professional & parent/other parent), 'normality' & identity
	"We mostly had him in the room. We always talked to him as if he were living. To talk to our son was the first step to the separation" (p26)	Relationship (parent/baby), 'normality', time, memory making & love
	"I didn't really look for outside support. We could talk about it a lot in the family. And we shared the grief and cried together" (p26)	Relationship (parent/family), acceptance

	"We had a wide circle of friend to call on. That was really great" (p26)	Relationship (parent/family/friends), acceptance
	"I believe Jesus brought us through this time…I don't know how we would have managed without our faith. We hope, through Jesus, that we'll see our daughter again" (p26)	Acceptance, spirituality
	"I found it really good to be in a course with other parents who'd had more or less the same experience. I can recommend that, it really helped" (p27)	Relationship (parent/other parent), 'normality'
	"We had time to prepare so that I could enjoy a second pregnancy and not panic before every scan. …in the first pregnancy we did our best and it didn't end well. But in the second pregnancy I accept it's not in our hands but in nature's and we have to leave it to nature" (p27)	Acceptance & Hope
	"The midwives even listened to the heartbeat. They treated me like a normal pregnant woman. And that gave me a good feeling instead of being pushed away because the baby would die" (p26)	Validation as someone who is pregnant
	"She prepared us for the birth so well, just we two and her. So really I had a normal birth preparation, what is labour, how does it go and [we] only later [talked] about our specific situation and what we could do" (p26)	Validation as someone who is pregnant & as a parent
	"For me, the exercise course was extremely important. Only that I went to something where every woman who'd given birth goes, to acknowledge that I'd given birth" (p27)	Validation as someone who has given birth
Cortezzo et al. (2019)	*''I felt like I was able to parent my daughter and advocate for her.'' (p1342)*	Relationship (parent/baby), sense of control, 'normality', identity
	''The entire team was on the same page''; and ''everyone worked well together.' (p1342)	Relationship (parent/professional), communication
	''It was good to know what to expect''; and ''we liked knowing what was going to happen.'' (p1342)	Relationship (parent/professional), communication & sense of control
	"...was not anticipating being in the moment of my child's death and wanting to do everything to save her even if I knew she was suffering...emotions for your sick child suffering in your arms is unbearable" (p1342)	Relationship (parent/baby), complex emotions & acceptance
	'I felt like I was able to parent my daughter and advocate for her'(p1342)	Validation as a baby

Perinatal meta-ethnography
Table S1

O’Connell et al. (2019)	*“The General Practitioner said that she didn’t know if she herself could go through with carrying a baby to term that would die. That scared me” (p14)*	Relationship (parent/professional), empathy (lack of), communication (lack of)
	“She [consultant] was so respectful, non- judgmental. I could not tell if she considered it a good idea to have a termination or not” (p14)	Relationship (parent/professional), communication, empathy
	“You couldn’t ignore the kicking anymore and she became ‘Holly’. We took her on trips to the beach and here, there and everywhere” (p15)	Relationship (parent/baby), memory making, time, normality
	“My toddler talked to baby Talia and would do face painting on my tummy for her” (p15)	Relationship (baby/sibling), memory making, time, normality
	“My husband would lie next to me so he could feel the kicks. You treasured them more because you knew that this was it” (p15)	Relationship (parent/baby), memory making, time, normality
	“I fell asleep holding her little foot and when I awoke it was warm as if she were alive and I had been dreaming about breast feeding her. It was really comforting but I thought, “I’ll never be able to give you the milk that is there for you” (p15)	Relationship (parent/baby), memory making, time
	“I wanted to shut everyone out, I didn’t want to explain it again and deal with their responses” (p15)	Relationship (parent/friends/family & parent/professionals), communication
	“Meeting another mother who had a baby with anencephaly and looked not only normal but actually good, was really reassuring to me”(p15)	Relationship (parent/other parent), empathy
	“During the scan, he [obstetrician] said: “there is nothing from here up”, as he indicated from his own brow, upwards, I couldn’t take it in” (p15)	Relationship (parent/professional), empathy (lack of), communication (lack of)
	“I had the sense that he [Consultant} considered my continuing the pregnancy a waste of his time and resources” (p15)	Relationship (parent/professional), empathy (lack of), communication (lack of)
	“She treated Talia like a human. Not like an unfortunate aberration which is the message I got elsewhere” (p15)	Relationship (parent/professional), communication, empathy

	"Everyone was supportive and sympathetic, the minute you walked into the hospital. It was as if they knew you your whole life. I couldn't have done it without them" (p15)	Relationship (parent/professional), communication, empathy
	"She sat with us and answered our questions kindly, sensitively and respectfully" (p15)	Relationship (parent/professional), communication, empathy
	"The midwife was lovely: I remember her stroking her [baby's] face and saying how beautiful she was"(p15)	Relationship (parent/professional & baby/professional), empathy, normality
	"Afterwards, I was still being called 'Mummy', that meant an awful lot to me" (p15)	To move on – love, new normality
	"I still had to get up for our other child, if we didn't it would have made carrying the baby ten times harder" (p15)	Relationship (parent/sibling)
	"That horrific internet picture is still in my mind" (p15)	Communication
	"I left the hospital with no information. It was just like a bad dream" (p15)	Relationship (parent/professional), communication
	"We took it moment by moment while she was with us and tried not to look into the mouth of the monster" (p15)	Sense of control
	"It was unbelievably painful but the most healing experience imaginable. She is a vibrant part of our family" (p15)	To move on – relationship (family), love, new normality
	"She had her father's dark hair and his earlobes, these things when times are dark bring me comfort" (p15)	To move on – relationship (baby/parent), love, new normality
	"I felt really protective of her, people did not need to know the nature of her condition" (p15)	Relationship (parent/baby), communication
	"She treated Talia like a human. Not like an unfortunate aberration which is the message I got elsewhere" (p15)	Relationship (parent/professional), communication, empathy
	"The midwife was lovely: I remember her stroking her [baby's] face and saying how beautiful she was"(p15)	Relationship (parent/professional), communication, empathy, normality

	"This baby's lifetime was while it was in the womb, my own flesh and blood, its journey was inside of me" (p14)	Relationship (parent/baby), love
	"We were respecting her life cycle, her time with us" (p14)	Relationship (parent/baby), love, time
	"He was getting bigger, more real and I started to appreciate every single day I had with him because that would be it" (p15)	Relationship (parent/baby), love, time, memory making
	"My husband would lie next to me so he could feel the kicks. You treasured them more because you knew that this was it" (p15)	Relationship (parent/baby), love, time, memory making
	"There's more to a baby than a head!"(p15)	Relationship (parent/baby), love
	"She's very much a fully formed baby to me and I didn't want anyone thinking of her as less than human or less as a baby" (p15)	Relationship (parent/baby), love, normality
	"I just knew I loved her" (p15)	Relationship (parent/baby), love, normality
	"Due to media coverage on how inhumane it was, to not offer termination in cases like mine, I began to feel like I was doing something wrong until I met another mother who said she had been so glad to have been able to meet and hold her baby"(p15)	Relationship (parent/baby), parent/other parent), normality
	"Afterwards, I was still being called 'Mummy', that meant an awful lot to me" (p15)	Relationship (parent/siblings), love
	"Everyone told me from day one that there was no hope but I always held onto that hope until the very minute up to the very end"(p15)	Relationship (parent/baby), love, hope
	" One look at our baby Alana, and you knew finally that she wasn't meant for this world"(p15)	Acceptance, love
	"It was unbelievably painful but the most healing experience imaginable. She is a vibrant part of our family" (p15)	Relationship (parent/baby/sibling), love, acceptance, normality
	"She had her father's dark hair and his earlobes, these things when times are dark bring me comfort" (p15)	Relationship (parent/baby), love
	"I find it had to use the language that Talia died [prematurely] because in a weird way it doesn't fit, she fulfilled her life cycle" (p15)	Acceptance, love

	"I think our lives improved immeasurably from having experienced Talia and the way she came to us" (p15)	Acceptance, love
	"It made me a better person a million times. Cillian has given me the gift to understand what I am about and what it means to be alive" (p15)	Acceptance, love
	"It was unbelievably painful but the most healing experience imaginable. She is a vibrant part of our family" (p15)	Acceptance, love, spirituality
	"This baby's lifetime was while it was in the womb, my own flesh and blood, its journey was inside of me" (p14)	Validation as a baby, validation as someone who was pregnant, validation as a parent
	"We were respecting her life cycle, her time with us" (p14)	Validation as a baby
	"He was getting bigger, more real and I started to appreciate every single day I had with him because that would be it" (p15)	Validation as a baby
	"You couldn't ignore the kicking anymore and she became 'Holly'. We took her on trips to the beach and here, there and everywhere" (p15)	Validation as a baby
	"My husband would lie next to me so he could feel the kicks. You treasured them more because you knew that this was it" (p15)	Validation as a baby, validation as a parent
	"She's very much a fully formed baby to me and I didn't want anyone thinking of her as less than human or less as a baby" (p15)	Validation as a baby
	"She treated Talia like a human. Not like an unfortunate aberration which is the message I got elsewhere" (p15)	Validation as a baby
	"The midwife was lovely: I remember her stroking her [baby's] face and saying how beautiful she was"(p15)	Validation as a baby
	"Afterwards, I was still being called 'Mummy', that meant an awful lot to me" (p15)	Validation as a parent
	"It was unbelievably painful but the most healing experience imaginable. She is a vibrant part of our family" (p15)	Validation as a baby, validation as parent
	"She had her father's dark hair and his earlobes, these things when times are dark bring me comfort" (p15)	Validation as a baby, validation as a parent

	"You can literally feel your heart breaking" (p14)	Complex emotions, loss of identity
	"I cried so much, I didn't know humans had reserves to cry that much" (p14)	Complex emotions
	"You have a little human being kicking the living daylights out of you and an unbearable pain of knowing there will be a void at the end" (p14)	Complex emotions
	"I've never experienced anything like that sheer panic and utter shock" (p14)	Complex emotions
	"We were in a daze, how could you carry a life that would not exist" (p14)	Complex emotions
	"I felt like a failure as a mother as well as bit of a freak" (p14)	Complex emotions
	"I felt like an absolute failure, I couldn't even have a baby" (p14)	Complex emotions
	"I didn't know who I was anymore or if I was ever coming back"(p14)	Complex emotions
	"You can take this thing out of me now, what's the point in being pregnant" (p15)	Complex emotions
	*"This baby is going to have no 'f******** head, I was freaked by that" (p15)*	Complex emotions
	"I am not a mother, this is not a baby. This thing has to go" (p15)	Complex emotions
	"I was growing a monster inside of me" (p15)	Complex emotions
	"What kind of a mother was I? I was afraid I would recoil when I saw her." (p15)	Complex emotions
	"I was so sorry for this girl it was happening to, I thought. "you poor thing," I was feeling sorry for myself outside of myself"(p15)	Complex emotions
	"Your whole body is saying, "where's the baby? " I needed to go down to the grave and get her. I just needed one more look at her" (p15)	Complex emotions
	"It's terrifying going again… a leap into the unknown. Oh my God, the courage to try and do it [become pregnant] again." (p15)	Complex emotions

Weeks et al. (2020)	*'everything had sort of been done... so... we were sort of presented with a few options and again, it was done very sensitively..... they sort of said, 'look, you know, if you want to take, take Elizabeth home, on a ventilator and we'll help you look after her at home with, you know, support and nursing care and whatever..... look at different things of, different ways of withdrawing treatment, whether you can do that in hospital um, or you can take her home and do that or'... they were... very understanding and ...very sensitive about it... there was one doctor in particular who sort of took us under his wing... I remember he said to us, that ... he'll basically do whatever we want to do as long as he doesn't have to go to jail, and (laughs) so I thought that was pretty, kind of him and in that he's just open to however we want to .....spend time with Elizabeth...' (p749)*	Relationship (parent/professional), communication, empathy, sense of control, time, memory making
	'[they] gave us the impression that... they were happy... very supportive... [about] the step that we'd taken to, to go ahead with it... gave us the impression that we were being brave about it... they were very supportive...' (p749)	Relationship (parent/professional), communication, empathy, sense of control,
	'I did find different specialities had completely different prognoses... which was quite confusing and confronting... and I remember going to [the hospital]... with the belief [she] had zero chance of survival... and then the surgeon... automatically went from zero to 80 percent which is really difficult to adjust to, and... [the] obstetrician that day... said, 'well I don't believe it's 80... at best, it's 50', so we had all these conflicting [opinions]... which was the most difficult thing...' (p749)	Relationship (parent/professional), communication (lack of)
	'I think it's important that families have choice... I think it's important that you're in control, and I don't think you can have professionals telling you what, obviously they need to guide you	Relationship (parent/professional), communication, sense of control

	and to some extent... but you need to feel like you are informed and that you have some sense of control...' (p749)	
	'the team approach... I couldn't emphasise that enough, if you don't have that team approach, and for the doctors to say... 'look, I don't know about the question you're posing', or this, 'I'm going to refer it and come back to you', [the obstetrician] being honest and open and up front, I, I really appreciated that...' (p749)	Relationship (parent/professional), communication, sense of control
	'[the obstetrician] was just amazing, he allowed me to hope... because if you don't have hope in a pregnancy like that... you won't cope' (p749)	Relationship (parent/professional), communication, empathy
	'there weren't many moments but it was one of those moments where you kind of almost felt normal and even though you're in the NICU, you kind of just block it all out... well this is us bathing our baby and you know, having time with our baby...' (p749)	Relationship (parent/baby), normality, time, memory making
	'they were always very sensitive, treated our child with respect... that was lovely... I can't speak more highly of the way the [hospital] looked after our Chloe, for those two months... I knew that [they] loved her, because they all cried when she (voice cracking) passed away, just like us.....they went way beyond the call of duty like, when she passed away, [the funeral directors] came to collect her, they even held her hand (voice cracking) as they walked around... that to me means a lot...' (p749)	Relationship (parent/professional & baby/professional), communication, empathy, love, dignity
	'you could see it, [the neonatologist] was clearly moved himself, he was very teary and I guess, it was like our family, or Alexandra's family..... they're really the only people who knew her, they, they were there grieving with us and then [when] she died... all the workers came in that morning, it was kind of just like an open house, you know, Alexandra was there, in this beautiful bassinet that they had made up for her... the social workers, the pastoral	Relationship (baby/professional & parent/professional), love

	care workers, the doctors, the nurses, the people who had cared for her...' (p749)	
	'they were always very sensitive, treated our child with respect... that was lovely... I can't speak more highly of the way the [hospital] looked after our Chloe, for those two months... I knew that [they] loved her, because they all cried when she (voice cracking) passed away, just like us.....they went way beyond the call of duty like, when she passed away, [the funeral directors] came to collect her, they even held her hand (voice cracking) as they walked around... that to me means a lot...' (p749)	Relationship (parent/professional & baby/professional), communication, empathy, love
	'[the obstetrician] was just amazing, he allowed me to hope... because if you don't have hope in a pregnancy like that... you won't cope' (p749)	Relationship (parent/professional), communication, empathy
	'everything had sort of been done... so... we were sort of presented with a few options and again, it was done very sensitively..... they sort of said, 'look, you know, if you want to take, take Elizabeth home, on a ventilator and we'll help you look after her at home with, you know, support and nursing care and whatever..... look at different things of, different ways of withdrawing treatment, whether you can do that in hospital um, or you can take her home and do that or'... they were... very understanding and ...very sensitive about it... there was one doctor in particular who sort of took us under his wing... I remember he said to us, that ... he'll basically do whatever we want to do as long as he doesn't have to go to jail, and (laughs) so I thought that was pretty, kind of him and in that he's just open to however we want to .....spend time with Elizabeth...' (p749)	Validation as someone who is pregnant, validation as a parent
	'they were always very sensitive, treated our child with respect... that was lovely... I can't speak more highly of the way the	Validation as a baby

	[hospital] looked after our Chloe, for those two months... I knew that [they] loved her, because they all cried when she (voice cracking) passed away, just like us.....they went way beyond the call of duty like, when she passed away, [the funeral directors] came to collect her, they even held her hand (voice cracking) as they walked around... that to me means a lot...' (p749)	
	'you could see it, [the neonatologist] was clearly moved himself, he was very teary and I guess, it was like our family, or Alexandra's family..... they're really the only people who knew her, they, they were there grieving with us and then [when] she died... all the workers came in that morning, it was kind of just like an open house, you know, Alexandra was there, in this beautiful bassinet that they had made up for her... the social workers, the pastoral care workers, the doctors, the nurses, the people who had cared for her...' (p749)	Validation as a baby
Crawford et al. (2021)	*"I guess that key points were just ultrasounds, always getting to know that he was alive and had a strong heartbeat. They recorded the ultrasounds for us so I have the video on a DVD of him actually alive. That was a big key point." (p406)*	Relationship (parent/baby), 'normality', memory making
	"When she [counselor] came, I felt like when she was there, I was safe. I felt so alone, except for when she was there. I feel like she knew what to do and what was happening and what was going on." (p408)	Relationship (parent/professional) and empathy
	"But they [counselors] were there when he was born, which meant that we did have somebody else who understood. One of my big worries was I really did not want a cute, little, happy student nurse popping in and congratulating me. Just go away and leave me alone because you just don't get it." (p408)	Relationship (parent/professional) and empathy
	"She had personal experience losing her child. And so that makes me trust and relate to her automatically more" (p408)	Relationship (parent/professional) and empathy
	"She came and took moulds of my baby's hands and feet and did imprints, and then she took pictures . . . So I love that. That was very	Relationship (parent/professional, parent/baby), memory making, love

	important to me, and she cut off a little piece of her hair, and that was helpful too just to have like an actual piece of her still." (p406)	
	"And even providing material things that you could have after the baby had passed away. That, I think, is an enormous, huge, huge thing because, especially for me, it was my first, and my body had showed all the symptoms of being a mother. I'd been pregnant for nine months, and then I had all the destruction from post-birth. It was very evident on my body, but I had no child in my arms. And to have something, to physically have something, to physically have a stuffed animal or photographs saying that my child lived, my child was here, that was a huge deal for me, and I was really thankful for those things that they provided." (p406)	Relationship (baby/parent, parent/professional), memory making, identity
	"And it made me really aware that our culture does not talk about death. Our culture does not like to talk about death, or we don't know how to handle it. We've lost track of— cultures all around the world have really specific rituals and ways in which they mourn, grieve, honor the deceased and departing loved ones. And we just don't." (p407)	Relationship (parent/family/friends)
	"[this other mother] had pictures of her baby on her front room walls. It was so awesome because I was getting worried that I was, I don't know, mourning too much about it, being like I want pictures of her in every room." By seeing how another mother incorporated her dead child's photographs into their family photo wall, this mother said she felt permission to do the same. "The fact that I saw her [photos on the wall] was [a message that] she still loved her babies, and she still cared about her babies." With that example, she decided, "I can do that with mine too. I don't have to act embarrassed that this thing happened and push it under the rug like it didn't happen. It happened" (p407)	Relationship (parent/other parent, baby/parent), acceptance & 'normality'
	"Hope is now hope for something better. Hope for a life after death. Hope that heaven is closer than we think. And I feel that and I know that" (p407)	Hope, acceptance, love, spirituality
	"My biggest hope was that she would change me, would change our family, and be a beacon of hope instead of something that was so painful." (p407)	Hope, acceptance, love, spirituality

	"And I just felt like that was my answer that I had been searching for, that my son was not meant to live. And actually, instead of bringing a sense of hopelessness to me, it brought a sense of, "Okay. This is what's supposed to happen, and now I can line up my behaviors accordingly," because that's what I wanted." (p408)	Hope, acceptance, love
	"And even providing material things that you could have after the baby had passed away. That, I think, is an enormous, huge, huge thing because, especially for me, it was my first, and my body had showed all the symptoms of being a mother. I'd been pregnant for nine months, and then I had all the destruction from post-birth. It was very evident on my body, but I had no child in my arms. And to have something, to physically have something, to physically have a stuffed animal or photographs saying that my child lived, my child was here, that was a huge deal for me, and I was really thankful for those things that they provided." (p406)	Validation as someone who was pregnant, as a parent & a baby
	"She came and took molds of my baby's hands and feet and did imprints, and then she took pictures . . . So I love that. That was very important to me, and she cut off a little piece of her hair, and that was helpful too just to have like an actual piece of her still" (p406)	Validation as a baby
	"[this other mother] had pictures of her baby on her front room walls. It was so awesome because I was getting worried that I was, I don't know, mourning too much about it, being like I want pictures of her in every room…The fact that I saw her [photos on the wall] was [a message that] she still loved her babies, and she still cared about her babies…I can do that with mine too. I don't have to act embarrassed that this thing happened and push it under the rug like it didn't happen. It happened" (p407)	Validation as a parent, validation as a baby
Hein et al. (2022)	*"It [Prenatal diagnostics] was lousy. What we experienced was absolutely and completely unacceptable. This was the worst of medical art. First she [prenatal diagnostician] says, she [the baby] has an incurable disease and will only survive a few days, if at all. Already five sentences later she says: We can remove her and then you try again. It was completely pathetic (. . .) It was the*	Relationship (parent/professional), empathy (lack of), communication (lack of)

	beginning of the whole story and at the same time the negative highlight." (p4)	
	"The situation was so bizarre. She [prenatal diagnostician] just left. She should have stayed. She should have told us, I see something suspicious. I want to take the time to explain this, and to provide information to help us understand, offer us a framework." (p5)	Relationship (parent/professional), empathy (lack of), communication (lack of)
	"We would have wished them to disclose all our options. They only said: 'Most parents in your situation decide to have an abortion'. They did not say: 'You can carry the pregnancy to term' and what we have to face afterwards, nobody told us" (p5)	Relationship (parent/professional), empathy (lack of), communication (lack of)
	"They told us the diagnosis and sent us home. They should have had something there (. . .) a room, to collect yourself, where you can recapitulate and think. What did just happen?" (p5)	Relationship (parent/professional), empathy (lack of), communication (lack of)
	"Before that we had an appointment with a human geneticist (. . .) This was not really instructive or helpful, I would say. She drew some kind of family trees of our parents and grandparents, how this is inherited. However, we already knew this because we did our own research after the diagnosis." (p5)	Relationship (parent/professional), empathy (lack of), communication (lack of)
	"However, in the end, this was the diagnosis and we were standing on the street, confronted with this thing, and thought, what now? I think, what we would have needed acutely, in the first moment, was somebody to collect us. Somebody to answer the first questions that spontaneously crossed our minds." (p5)	Communication and sense of control
	"Our children came (. . .) And we went for a walk. I had a basket (. . .) There we put her and covered her. Then we went with our three children to a playground near the hospital. We made some pictures, how they played. And I was sitting on a rocker with the basket beside me and thought, what would people say if they knew	Relationship (parent/baby/siblings), memory making, time

	there is a dead baby in the basket. Crazy, right? But this is family life, right?" (p10)	
	"I wrapped her [deceased daughter] up and we went directly home with her, one and a half hours. Exactly. We just held her and went home in our car (. . .) The only thing was that the hospital administration called us the next day and told us the baby was missing. They said they would sent us the police, because we are not allowed to do this. And we said, okay, we will lock the door. The police will not enter and take away our dead child" (p10)	Relationship (parent/professional), communication (lack of), empathy (lack of), time, memory making
	"The burial was on Wednesday. We had her at home until then. We had her in a dark and cool room and got her out once a day in the evenings. We took her to the living room and spent time with her. We sat with her, held her, sang to her. We took pictures. Family pictures on the couch with a self-timer" (p10)	Relationship (parent/baby), time, memory making, love, sense of control, normality
	"This was my baby. She was in my belly. She was kicking. She was happy (. . .) and it was like: Whoa! This would be murder (. . .) I cannot do this. I do not want to. I will not." (p6)	Acceptance, love
	"If the child wants to die, it will die on its own. I am not the one who has the right to decide whether the child will live or die (. . .) I will not do this. Let her decide on her own" (p6)	Acceptance
	"Then, we just celebrated her birthday each day (. . .) There was cake and ice cream every day" (p9)	Relationship (baby/parent), acceptance, love, memory making, normality
	"I did not expect it, but it was important for us to have her home, because you could clearly see how the soul leaves the body. Well, you have seen the child alive before and you have seen it dead one day later, which is not very pleasant, to put it politely. However, as we saw the body and could sense and see and feel how the soul leaves the body, that nobody is home any more in that little body. Seeing this in the course of these days allowed the own soul, the	Relationship (baby/parent), acceptance

	own spirit and mind, the own heart to come along, to comprehend that she has died". (p9-10)	
	"The burial was on Wednesday. We had her at home until then. We had her in a dark and cool room and got her out once a day in the evenings. We took her to the living room and spent time with her. We sat with her, held her, sang to her. We took pictures. Family pictures on the couch with a self-timer" (p10)	Relationship (baby/parent), acceptance, love, memory making, normality
	"Then, we just celebrated her birthday each day (. . .) There was cake and ice cream every day" (p9)	Validation as a baby
	"Our children came (. . .) And we went for a walk. I had a basket (. . .) There we put her and covered her. Then we went with our three children to a playground near the hospital. We made some pictures, how they played. And I was sitting on a rocker with the basket beside me and thought, what would people say if they knew there is a dead baby in the basket. Crazy, right? But this is family life, right?" (p10)	Validation as a family
	"The burial was on Wednesday. We had her at home until then. We had her in a dark and cool room and got her out once a day in the evenings. We took her to the living room and spent time with her. We sat with her, held her, sang to her. We took pictures. Family pictures on the couch with a self-timer" (p10)	Validation as a baby, validation as a parent
	"You leave the examination room. You stay in the corridor. In front of the registration desk. At some point, they return you the maternity logbook. You could cry or you compose yourself. People either look at you or ignore you. As if you weren't there. You don't know what to do with yourself. You cannot entrench yourself forever in the toilet. You are completely confused. Yet you have to make it outside and see how you manage to continue" (p4)	Complex emotions

	"I left the place surrounded by a fog. I knew my child is not doing well. But I was feeling all right. She was moving. My feelings were actually good. I left the place completely confused. I did not know what was happening. Then I called my husband and cried. I said: ‚I do not know anything. My child. Either she is severely disabled or she is doing fine.' It seems like a mix-up. Honestly, I did not know anything anymore." (p5)	Complex emotions
	"I think, whatever happens, even an unborn child does not have the right to blow up a whole family (. . .) Just imagine, a handicapped child is born and the family falls apart (. . .) This would be very bad." (p6)	Complex emotions
	"It left marks on everybody. Our oldest daughter became vegetarian. She was 13 years old and said, she will never have children on her own. She doubted her faith because of what God puts us through (. . .) Our next daughter had a breakdown after three years. Her subconscious had been dealing with death the whole time (. . .) She was afraid to go to bed one day and not wake up the next day (. . .) My next daughter suddenly had back pain, back pain, back pain (. . .) One of my sons became very aggressive (. . .) The youngest had a (. . .) severe peanut allergy, which is life-threatening (. . .) now he knows, death is definitive. So he stopped eating at school. He refused snacks at school, because he was afraid." (p11)	Relationships (baby/family), complex emotions
	"I did not expect it, but it was important for us to have her home, because you could clearly see how the soul leaves the body. Well, you have seen the child alive before and you have seen it dead one day later, which is not very pleasant, to put it politely. However, as we saw the body and could sense and see and feel how the soul leaves the body, that nobody is home any more in that little body. Seeing this in the course of these days allowed the own soul, the	Acceptance, hope, love, spirituality

	own spirit and mind, the own heart to come along, to comprehend that she has died". (p9-10)	
